# Sustainable Materials Design With Multi‐Modal Artificial Intelligence

**DOI:** 10.1002/advs.202524273

**Published:** 2026-04-15

**Authors:** Tianyi Xu, Tianshuo Wei, Yan Ge, Bo Peng, Yue Li, Maolin Wang, Peng Wen, Chao Yang, Ye Wei

**Affiliations:** ^1^ Department of Data Science City University of Hong Kong Hong Kong China; ^2^ Department of Materials Science City University of Hong Kong Hong Kong China; ^3^ School of Materials Science and Engineering Shanghai Jiao Tong University Shanghai China; ^4^ Inner Mongolia Research Institute Shanghai Jiao Tong University Hohhot China; ^5^ Department of Mechanical Engineering Tsinghua University Beijing China; ^6^ State Key Laboratory for Advanced Metals and Materials University of Science and Technology Beijing Beijing China; ^7^ Hong Kong Institute of AI for Science City University of Hong Kong Hong Kong China

**Keywords:** AI‐driven material discovery, multi‐modal AI, sustainable materials

## Abstract

The growing scarcity of critical minerals, coupled with high embodied carbon emissions and persistent pollution from material smelting, highlights the urgent need for a sustainable transformation in materials design. This challenge can be approached as a complex multi‐objective optimization problem, requiring the simultaneous consideration of performance, economic viability, recyclability, and full life‐cycle environmental impacts. However, the conventional methodologies are increasingly strained by the exponential growth of heterogeneous, high‐dimensional data, which significantly constrains their optimization performance in complex engineering scenarios. In response, multi‐modal artificial intelligence (AI) offers a transformative pathway by enabling accelerated, data‐driven materials design through the integration of diverse textual, visual, and temporal information, thereby efficiently identifying compositions and structures that meet functional and sustainability criteria. This review synthesizes advances across six themes: multi‐modal AI foundations for learning composition–processing–structure–property–sustainability relationships; AI‐driven sustainable alloy discovery; autonomous laboratories with life‐cycle feedback; recyclable and reusable material design; AI‐optimized alloys for renewable energy and carbon capture; and data integration challenges, culminating in a roadmap that couples interoperable data infrastructures, human‐in‐the‐loop validation, and autonomous experimentation to accelerate equitable, sustainable materials discovery at scale.

## Graphical abstract

Critical mineral scarcity, high embodied carbon, and persistent pollution from materials processing intensify the need for sustainable materials design. This review frames the problem as multi‐objective optimization under heterogeneous, high‐dimensional evidence and highlights multi‐modal AI as an enabling pathway. Advances are synthesized across foundations, sustainable alloy discovery, recyclable and reusable materials, autonomous laboratories with life cycle feedback, and materials for energy and carbon capture, culminating in a verification oriented roadmap.

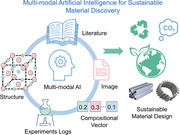

## 1 Introduction

**FIGURE 1:**
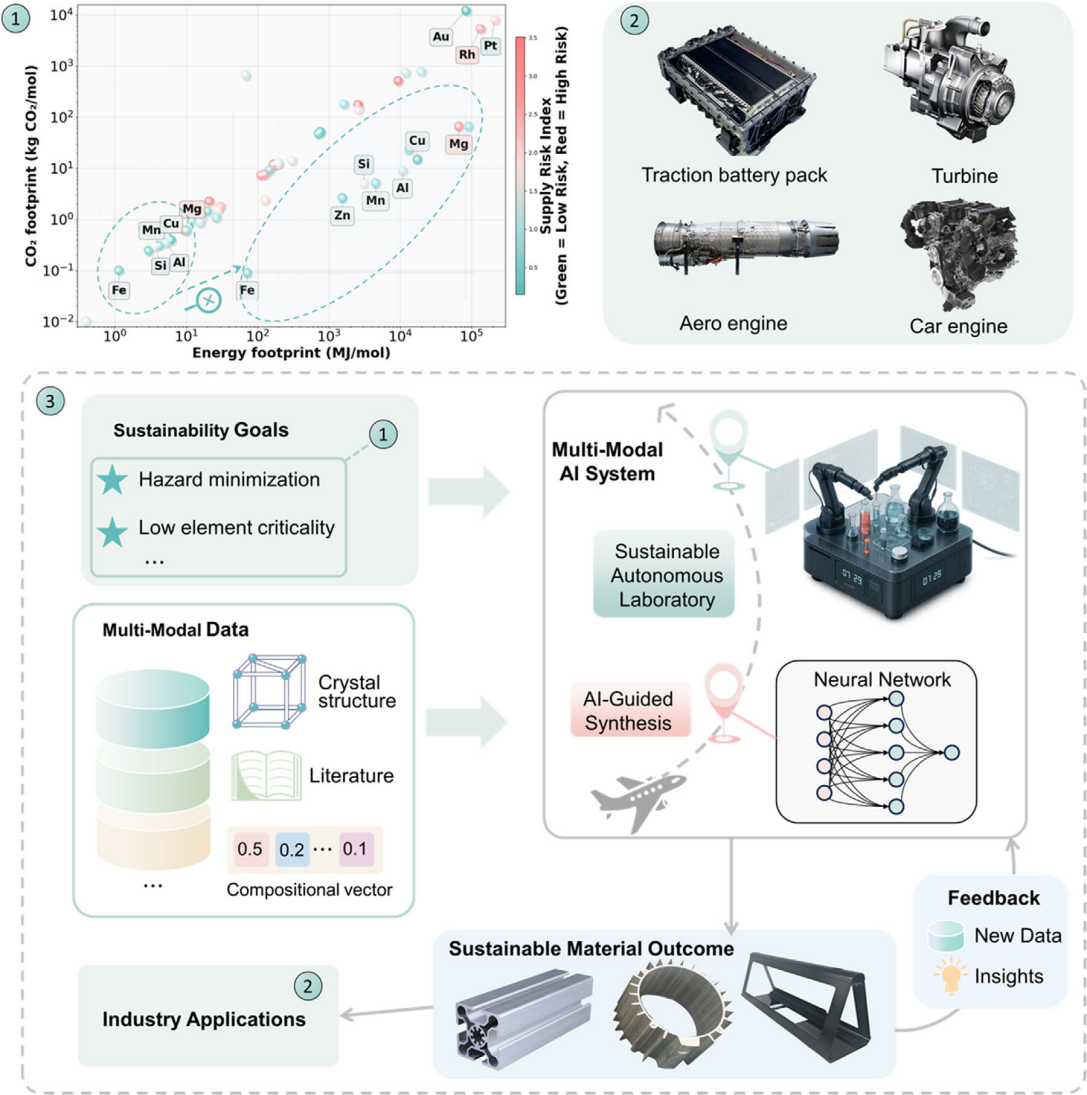
Multi‐modal AI framework for sustainable materials. (1) Elemental sustainability map: log–log scatter of energy footprint (MJ ${\mathrm{mol}}^{-1}$) versus ${\mathrm{CO}}_{2}$ footprint (kg ${\mathrm{CO}}_{2}$ ${\mathrm{mol}}^{-1}$), which is also shown in [20, 21]. Point color encodes a composite supply‐risk index (green = low, red = high) derived from supply concentration (USGS‐based HHI [22]) and country‐level ESG [23], which is consistent with [21]. (2) Representative industrial contexts: traction battery pack, turbine, aero engine, car engine. (3) Toward autonomous labs: evolving the pipeline from AI‐guided synthesis to a sustainability‐aware, closed‐loop *autonomous laboratory* that iteratively selects experiments, executes them robotically, and updates models via feedback.

Human society is confronting an unprecedented series of global crises that fundamentally demand a profound revolution in the field of materials science, an area nonetheless acclaimed as a pillar of the technological revolution and a pioneer in interdisciplinary innovation [1, 2]. First is the issue of resource depletion, particularly the increasingly strained supply of critical minerals such as cobalt and rare‐earth elements, which not only elevates economic costs but also incites geopolitical tensions [3, 4, 5]. Second is climate change; the production, processing, and transportation of materials are major contributors to global greenhouse gas emissions, and the “embodied carbon” of materials has become a critical consideration in achieving carbon neutrality targets [6, 7, 8] (Figure 1 (panel 1)). Thirdly, escalating environmental pollution, exemplified by acidic gases and heavy metal fumes in waste gas, as well as heavy metals in wastewater and solid waste, is inflicting irreversible damage upon global ecosystems [9, 10]. Last but not least, the development of cutting‐edge technologies such as information, biomedicine, and energy has continuously expanded the requirements for material properties. It is necessary to optimize material properties across multiple dimensions, and identifying new materials that can accurately meet structural, functional, and production needs has become the key to interdisciplinary innovation. Under the premise of sustainable development, optimizing the design of material compositions to meet diverse performance requirements has become an inevitable trend in the development of materials science.

Historically, materials discovery has been predominantly empirical, driven by design rules distilled from accumulated experimentation rather than derived purely from first principles. *Kao* *Gong Ji* [11], a seminal Chinese treatise compiled during the Zhou dynasty (mid‐first millennium BC), provides one of the earliest recorded examples of composition–application relationships in materials practice. It records six bronze recipes spanning compositions from approximately five parts copper and one part tin to near equal‐part mixtures, illustrating an early link between alloy composition and intended application. These recipes provide one of the earliest recorded examples of linking alloy composition to intended function and application.

This paradigm evolved significantly in the modern era. In 1781, Bergman identified the role of carbon in distinguishing wrought iron, steel, and cast iron, while Sorby's microscopic examination of steel in 1863 laid the foundations of metallography and helped reveal the importance of microstructure in determining material behavior. Subsequent advances in microscopy and analytical techniques enabled the precise determination of composition and structure across scales, establishing the composition–structure–property framework that defines modern materials science. By the mid‐twentieth century, computational methods such as PHACOMP began shifting the field from trial‐and‐error toward knowledge‐based exploration, enabling the screening of superalloys to suppress detrimental phases [12, 13]. However, the intrinsic complexity of materials data, which is naturally multi‐modal, encompassing composition, processing, and microstructure, remains a formidable challenge.

Despite these methodological advancements, the traditional discovery cycle remains ill‐equipped to meet current urgencies. The trajectory from laboratory proof‐of‐concept to market application is often measured in decades, creating a severe bottleneck in the pursuit of sustainable development [14, 15] (Figure 1, panel 2).

Consequently, sustainability in materials discovery should not be understood as a single performance metric, but rather as a broader framework. In this review, we use the term in an inclusive sense that covers more resource‐efficient research and development through reduced computational, experimental, and material costs, the incorporation of environmental impact and resource considerations into optimization, the discovery of advanced materials for sustainability‐relevant applications such as carbon capture, renewable energy storage, and green catalysis, the improvement of durability, degradation resistance, and service lifetime to reduce waste, and the promotion of circularity through recyclability, scrap tolerance, and end‐of‐life management. This broad perspective serves as a conceptual lens for the review and helps clarify which aspect of sustainability is being addressed in the different methodologies and case studies discussed below [16, 17, 18, 19].

To address the aforementioned challenges, data‐driven science is emerging as a central approach for the discovery of next‐generation materials, complementing the established eras of experiment, theory, and computation [24, 25, 26]. Mainstream computational approaches, exemplified by density functional theory (DFT) [27, 28], have enabled the prediction of ultra‐strong magnets, superconductors, and other functional materials [29, 30]. However, their cost and scope highlight the limitations of conventional computation [31, 32]: interatomic interactions are complex, the combinatorial space of possible chemistries is enormous (the composition space grows combinatorially even for quaternary systems) [33, 34, 35], and most calculations are performed for ordered crystals under idealized conditions near absolute zero [36, 37, 38]. Real materials, by contrast, fundamentally obey the laws of physics but often exhibit immense complexity, such as structural disorder and dynamic behaviors under varying temperatures and pressures [36, 39, 40]. Furthermore, the successful synthesis and design of these materials have historically relied on tacit human heuristics, such as empirical rules for elemental doping or phase stability, which are difficult to encode into explicit physics‐based equations [14, 41, 42]. As a result, conventional computation tends to explore only a tiny, biased subset of the design space and can easily converge to suboptimal solutions, especially for highly constrained structures such as periodic inorganic crystals [33, 43]. Using artificial intelligence (AI) to search for new materials across vast datasets can break through brute‐force screening bottlenecks, expand the exploration scope [1, 44], uncover tacit structure–property laws that improve design accuracy, and ultimately support closed‐loop optimization that connects experiments with applications [2, 45]. The integration of AI, particularly multi‐modal AI, signifies the deepening and evolution of this paradigm [46, 47, 48, 49]. It is not merely an accelerator of existing processes but an enabler of entirely new research and development modalities [50]. The core of this transformation lies in shifting materials design from a traditional “forward” process (i.e., synthesizing a material and then characterizing its properties) to a more efficient and purposeful “inverse design” framework (i.e., first defining a desired set of properties and functions, and then reverse‐engineering the material structures capable of meeting these requirements) [14, 51, 52, 53].

The implications of this shift are far‐reaching. Traditional discovery has relied on intuition and chance, probing only the neighborhood of known chemistries [29]. By contrast, modern AI paradigm such as generative models, can learn distributions over existing materials data and propose novel, sometimes counter‐intuitive candidates [54, 55, 56], thus enabling systematic exploration of design spaces whose scale is unreachable with conventional experiment or computation [57]. Crucially, the exploration can be goal‐driven: targets include not only functional performance but also constraints on embodied energy and environment (Figure 1, panel 1) and application‐specific requirements (Figure 1, panel 2), turning discovery into a multi‐objective optimization problem [58, 59].

However, data in materials science are inherently multi‐modal [24]. A single material system can generate microscopy and tomography images, diffraction and spectroscopy patterns, time‐resolved measurements, mechanical, thermal, and electrochemical tests, as well as process logs and textual reports [60, 61]. This diversity reflects the need to comprehensively understand, tailor and optimize materials across hierarchical length scales, diverse properties and dynamic application scenarios [62, 63]. Here we use “multi‐modality” to denote the principled fusion of such heterogeneous evidence, including crystal and microstructural representations, images, spectra, text corpora and tabular or numeric measurements, into coherent AI systems [47, 64, 65, 66, 67]. Such integration is essential to learn across the full materials lifecycle and to embed sustainability constraints directly into the design process [68]. In practice, this enables a progression from AI‐guided synthesis (human‐in‐the‐loop) to self‐driving, sustainability‐aware autonomous laboratories that close the loop between design, make, and test via active learning and robotic execution (Figure 1, panel 3) [2, 45, 69].

This paper aims to explore the current landscape, challenges, and future prospects of employing multi‐modal AI methodologies in the pursuit of sustainable materials [46]. Specifically, this review explores the transformative role of multi‐modal AI in Sustainable Materials Design, highlighting its ability to overcome the limitations of traditional unimodal AI approaches. By integrating diverse data sources, such as computational simulations, experimental results, imaging data, and scientific literature, multi‐modal AI enables the rapid discovery and optimization of materials with tailored properties, including recyclability, biodegradability, and energy efficiency [55, 57]. Beyond conventional methods, AI‐driven models predict material performance and environmental impact, optimizing production processes to reduce reliance on resources and minimize environmental pollution [70, 71].

## 2 Foundations of Multi‐Modal AI for Materials Design

The rapid development of modern materials discovery increasingly relies on multi‐modal data integration [1, 46, 47]. While many advances have been achieved using single‐modality approaches, such as structure–property correlations [66, 72] or image‐based defect analysis [73, 74], the growing diversity of data sources now enables a more comprehensive, cross‐scale understanding of materials behavior [24, 68]. This section outlines the foundations of multi‐modal AI, which seeks to unify information from distinct modalities to accelerate materials discovery [64, 75].

**FIGURE 2:**
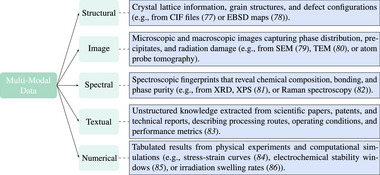
Illustration of multi‐modal data sources in materials discovery.

**FIGURE 3:**
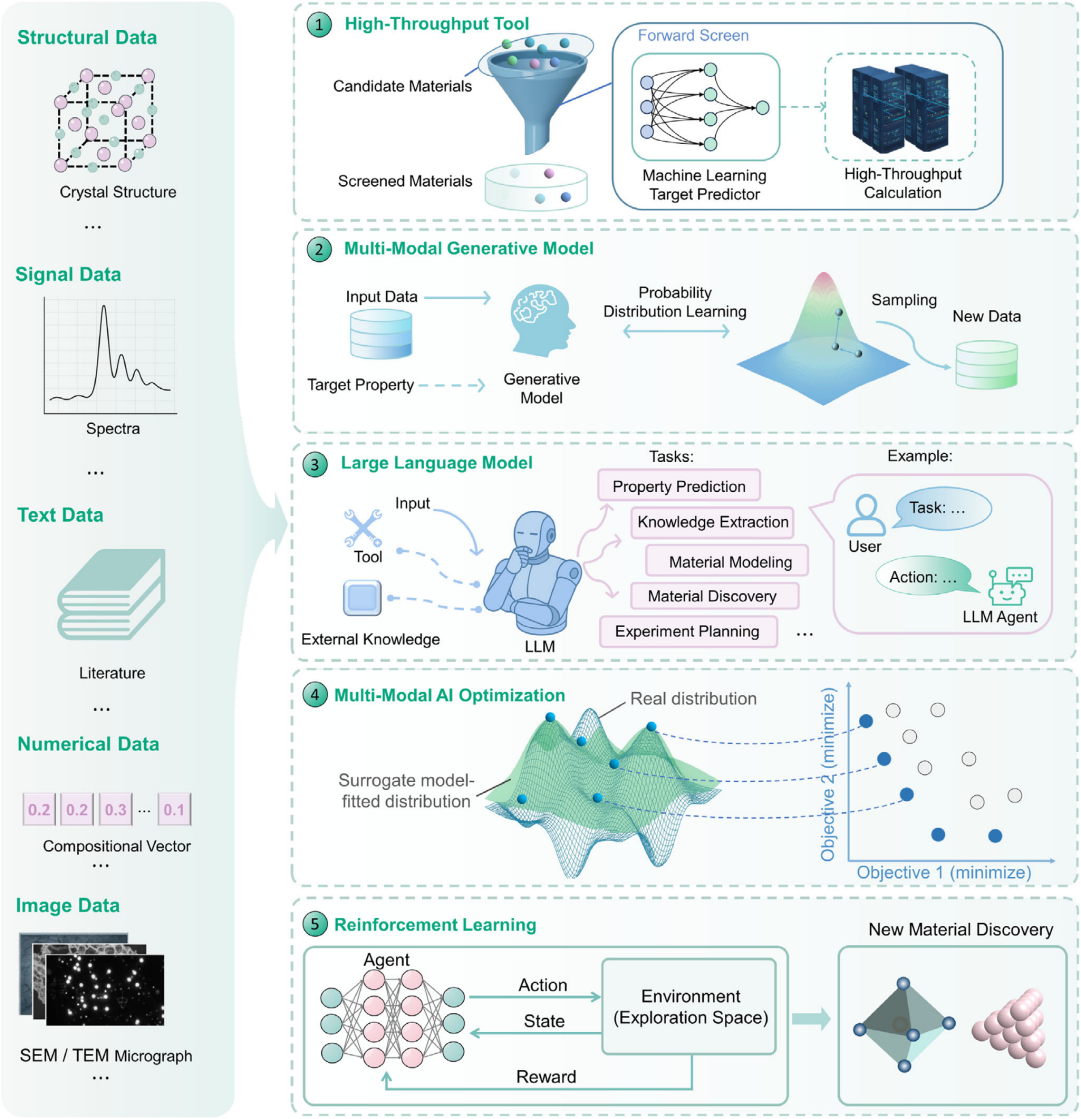
Foundations of multi‐modal AI for materials discovery. Five complementary paradigms illustrate how multi‐modal data (left) interface withand flow intomodern AI methodologies (right): (1) *High‐throughput tools*: machine learning predictors rapidly screen candidates. (2) *Multi‐modal generative models*: learn distributions and sample material representations. (3) *Large language models*: handle multi‐tasks such as property prediction, knowledge extraction and experiment planning. (4) *Multi‐modal optimizations*: fit surrogate models to heterogeneous data and perform multi‐objective optimization. (5) *Reinforcement learning*: sequential decisions in chemical space; optimise via rewards.

Figure 2 summarizes the principal data modalities underpinning multi‐modal learning, including structural, image, spectral, textual, and numerical data [47, 76]. Together, these modalities capture different aspects of the materials lifecycle. Building on these data foundations, Figure 3 presents the organizing framework used in this review, linking these modalities to five complementary AI paradigms in multi‐modal materials informatics. Table 1 provides a compact overview of the main paradigms in sustainability‐oriented materials design by summarizing representative method families, the modalities they leverage, typical sustainability targets, and their key advantages and limitations. These comparisons provide a useful lens for the remainder of the review. The following sections discuss how different paradigms balance cost, fidelity, novelty, grounding, sample efficiency, and validation burden. In this context, high throughput screening and machine learned interatomic potentials are mainly associated with the balance between cost and fidelity, generative models with novelty, coverage, and constraint satisfaction, large language model and agent‐based workflows with interpretability, grounding, and robustness across tool chains, and optimization and reinforcement learning with sample efficiency and validation cost. The Challenges section then summarizes common failure modes and possible verification centered mitigation strategies.

**TABLE 1:** Overview of multi‐modal AI paradigms for sustainability‐oriented materials design.

Method family	Modalities	Sustainability targets	Advantages	Limitations	Refs.
ML‐assisted LCA proxies	T, Tab, Sim	Carbon / energy / toxicity proxies (often production‐stage)	Rapid early screening; scalable hotspotting; ML completion of missing LCI fields	Not a full ISO LCA; boundary/FU/allocation sensitive; limited use/EoL coverage; harmonization + UQ / OOD issues	[87, 88, 89]
Multifidelity surrogates + active learning	S, Sim	Fewer costly DFT / experiments	Cost‐aware querying; reduces high‐fidelity evaluations	Needs cross‐fidelity correlation + calibration; UQ quality critical	[90, 91]
ML force fields (MLIPs) for atomistic simulation	S, Sim	Stability / durability / adsorption‐related tasks	Often near‐DFT accuracy in‐domain; fast geometry opt. + MD / MC; transferable via fine‐tuning	Coverage‐limited; OOD failures; validation/retraining needed for quantitative use	[92, 93, 94, 95]
Generative models for inverse design	T, S	Energy storage / catalysis / carbon capture; constraint‐/criticality‐aware design	Constraint‐guided generation; narrows search space	Requires DFT / experiments; imperfect constraints; bias/OOD risks	[96]
LLM / agentic workflows (RAG + tool use)	T, Tab, Code	Constraint‐aware planning; experiment / simulation orchestration	RAG‐supported constraint extraction; tool‐augmented workflows	Hallucinations; corpus bias; brittle tool‐chains; reproducibility challenges	[97, 98, 99, 100]
AI‐driven optimization	S, P, Sim	Resource‐efficient multi‐objective trade‐offs (incl. footprint proxies)	Bayesian / MOO search; Pareto + constraints; couples CALPHAD / DFT / experiments	Sensitive to objectives/constraints; validation remains costly; model mismatch risks	[101, 102, 103]
Reinforcement learning (sequential design)	S, P, Sim	Goal‐driven policies (reward can include sustainability terms)	Learns sequential policies; handles multi‐step constraints; simulator / platform compatible	Reward design hard; sample‐hungry; sim‐to‐real gap; safety constraints needed	[104, 105, 106]
Self‐driving laboratories (closed‐loop experiments)	T, I, P, Sim	Reduced trial‐and‐error; reproducible workflows	Autonomous execution + adaptive next‐run selection	High setup / safety burden; sustainability metrics incomplete without LCA‐aware objectives	[2, 107]

*Abbreviations*: T = text / documents; Tab = structured tabular data; I = images (e.g., micrographs); S = material structure (crystal / molecular / atomic configurations); P = process/recipe variables; Sim = simulation outputs; Code = executable code and tool traces. LCA = life cycle assessment; ISO = International Organization for Standardization (ISO 14040 / 14044 LCA framework); LCI = life cycle inventory; FU = functional unit; allocation = co‐product burden splitting; EoL = end‐of‐life stage; UQ = uncertainty quantification; OOD = out‐of‐distribution generalization; DFT = density functional theory; MLIP = machine‐learning interatomic potential; MD = molecular dynamics; MC = Monte Carlo simulation; LLM = large language model; RAG = retrieval‐augmented generation; CALPHAD = CALculation of PHAse Diagrams.

### 2.1 Multi‐Modal AI as a High‐Throughput Screening Tool

**FIGURE 4:**
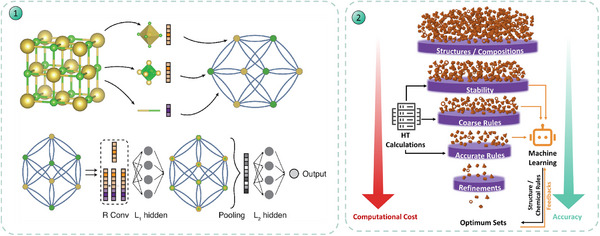
High‐throughput screening with AI. (1) Crystal graph convolutional neural network (CGCNN) for material prediction and screen. Adapted with permission from ref. [66], American Physical Society. (2) Funnel‐type workflow illustrates sequential filtering of candidate structures balancing computational cost and accuracy. Adapted with permission from ref. [108], Wiley Online Library.

In contemporary materials design, high‐throughput screening (HTS) typically adopts a cost–accuracy funnel (Figure 4, panel 2). Large candidate spaces are iteratively narrowed through stability checks, low‐cost electronic estimates, learned surrogates, and high‐fidelity evaluations [14, 29, 108]. To inform this stage‐wise filtering, HTS increasingly integrates multi‐modal data. A central design choice is structural representation; for example, crystal‐graph neural networks (CGCNN) encode atoms and bonds to produce interpretable crystal‐level embeddings for accurate property prediction (Figure 4, panel 1) [66]. This is often implemented as a multi‐fidelity funnel: low‐cost surrogates or approximations triage candidates before targeted high‐fidelity verification.

Beyond property predictors, machine‐learning interatomic potentials (MLIPs) provide fast approximations to DFT‐level energies and forces, enabling rapid geometry relaxation and large‐scale atomistic simulation within screening pipelines. Recent foundation MLIPs such as CHGNet [92], MACE‐MP‐0 [93], and Orb [94] aim to generalize across broad chemistry spaces, making them attractive for high‐throughput evaluation of stability, durability‐related energetics, and adsorption / reaction‐relevant configurations when used with appropriate validation. At the same time, their accuracy remains coverage‐dependent and can degrade in underrepresented regimes (e.g., defects, surfaces, and rare events), so sustainability‐critical conclusions should still be paired with uncertainty checks and targeted high‐fidelity verification.

Beyond structure, modern HTS pipelines leverage heterogeneous evidence streams to drive gating and sampling decisions. Image encoders extract microstructural descriptors from electron microscopy to capture phases and processing history [58, 73]. Simultaneously, literature mining provides priors on synthesis routes and property trends [109, 110], while spectral and diffraction signals are integrated with learned models to guide experimental selection in closed‐loop campaigns [111, 112, 113, 114, 115, 116].

This forward‐screening paradigm has been successfully applied across diverse domains. Pipelines utilize convex‐hull and vibrational analyses to identify stable compounds [117], screen electronic descriptors for optoelectronics [108], and discover ultralow thermal conductivity half‐Heuslers [118] alongside computational screening of potentially exfoliable 2D magnetic materials, including candidate ferromagnets with estimated Curie temperatures from model calculations [119]. Despite these successes, forward HTS remains constrained by the distribution of existing databases and severe class imbalance, often expending vast computation on negative candidates. These limitations motivate the shift toward generative models and large language models to explore beyond known distributions, alongside active and reinforcement learning frameworks that cast materials design as sequential decision‐making under uncertainty [51, 55, 104, 120].

### 2.2 Multi‐Modal Generative Models

Generative models such as generative adversarial networks (GANs) [122, 123, 124], variational autoencoders (VAEs) [125], and recent diffusion models [126, 127] are increasingly used for materials discovery, especially for inverse design where models sample candidates from a learned data distribution under conditioning signals rather than enumerating templates [128]. Incorporating multi‐modal data, such as structures, lattices, compositions and text, pushes this line further by enabling conditioning across chemistry, symmetry, and scalar properties within a generative framework [121, 128]. However, such generators remain fundamentally bounded by the support of their training data; in practice, inverse design should be viewed as proposal generation that must be coupled with uncertainty awareness, constraints, and downstream verification, especially near distribution boundaries [129].

**FIGURE 5:**
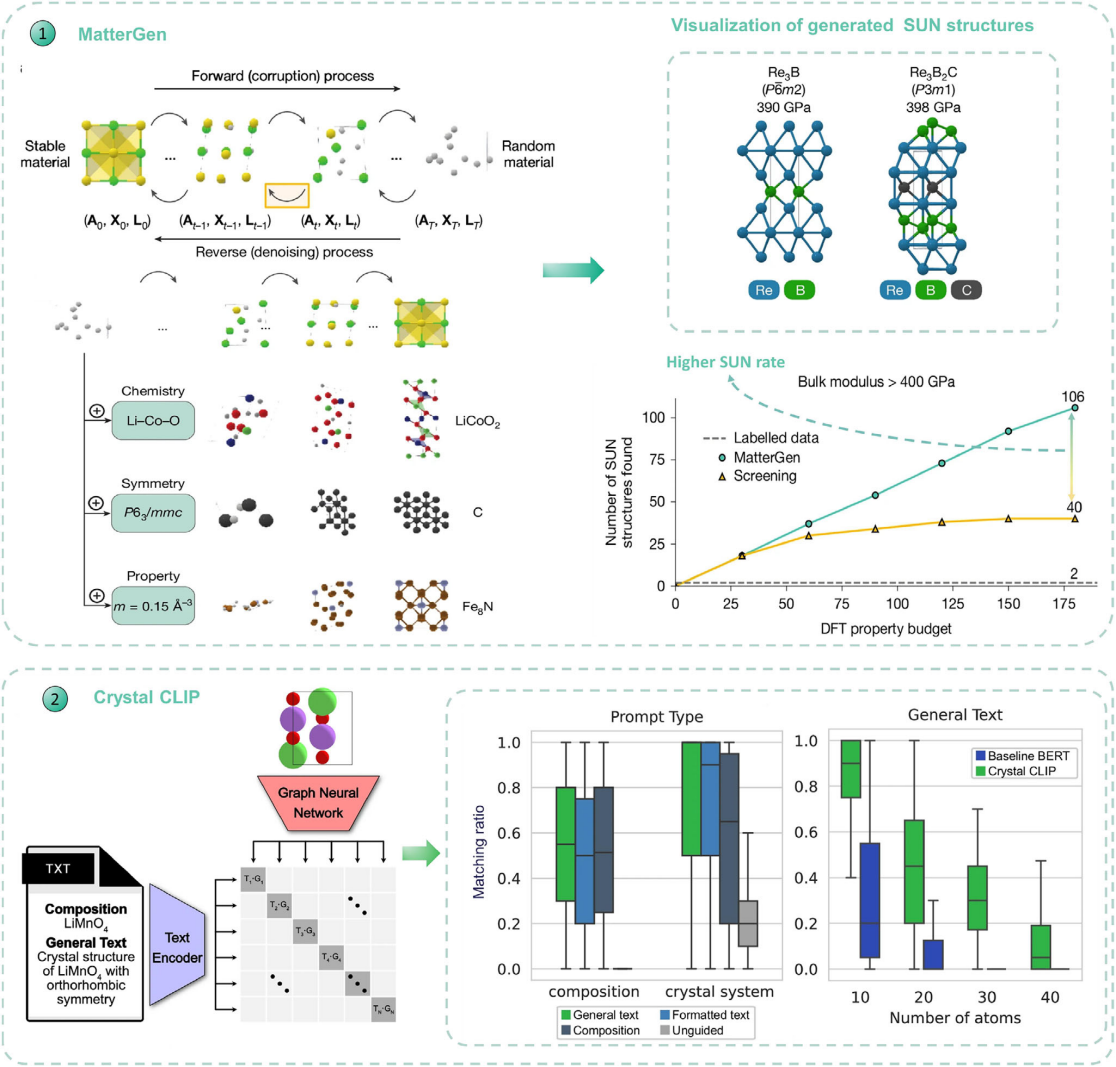
Generative model for material design. (1) MatterGen generates stable, unique, and new (SUN) crystals by reversing a diffusion process on atom types, coordinates, and lattices. Reprinted from ref. [96], CC BY 4.0. (2) The effectiveness of using multi‐modal data. Chemeleon integrates contrastive alignment between text and crystal‐graph embeddings with a text‐conditioned diffusion generator, enabling prompt‐guided crystal generation and improving composition and crystal‐system matching. Crystal CLIP conditioning markedly improves composition matching across atom‐count ranges and enhances both composition and crystal‐system matching ratios relative to unguided or baseline text prompts, confirming the effectiveness of multi‐modal conditioning in generative materials design. Reprinted from ref. [121], CC BY 4.0.

Compared with conventional methods, generative models can potentially offer the higher hit‐rate of stable–unique–novel (SUN) candidates, by directly sampling candidates from learned distribution. As shown in Figure 5 (panel 1), by jointly denoising atom types, coordinates, and lattices, MatterGen achieves obvious higher SUN rate than screening [96]. Conditioning signals (e.g., target properties, symmetry, or compositional constraints) can be integrated into crystal generators to steer sampling and reduce post‐hoc rejection [130, 131, 132]. For example, the panel (2) of Figure 5 shows that Crystal CLIP, which aligns text embeddings with crystal‐graph embeddings, shows substantially higher composition‐matching in text‐guided generation than a baseline BERT encoder, demonstrating effective text‐to‐crystal conditioning. Furthermore, Objectives can also be incorporated during sampling to reduce post‐hoc rejection. In particular, property‐guided or symmetry‐aware sampling steers generation toward target thresholds. MatterGen supports fine‐tuning with lightweight adapters for chemistry, symmetry, or scalar properties, and property‐guided sampling shifts the distribution of SUN samples toward these targets while outperforming substitution or random‐structure search under matched DFT budgets [96]. Exemplar SUN structures under such guidance are visualized in Figure 5 panel (1). Finally, by replacing hand‐crafted, modular templates with learned distributions, generative models help mitigate pipeline bias and potentially uncover less‐anticipated, eco‐friendly chemistries [96, 123].

**OOD Risk and “Propose–Verify” Framing**. While generative models can efficiently explore candidate spaces, their reliability is fundamentally limited by the coverage of training data and can degrade under distribution shift, especially near under‐sampled regions involving new chemistries, defects/surfaces, or processing‐dependent microstructures [133]. Accordingly, we view inverse design as *proposal generation* rather than a guarantee that the learned latent space faithfully encodes the governing physical laws, and emphasize coupling generation with (i) epistemic uncertainty awareness, (ii) executable physical / chemical constraints, and (iii) verification via simulation or experiment [96, 130]. This propose–verify perspective is especially important when sustainability constraints are system‐level, non‐differentiable, and context‐dependent (e.g., LCA choices such as functional units and system boundaries) [134]. We discuss OOD generalization and verification‐centric mitigation strategies in more detail in Section 7.2.2. We also briefly note that improving robustness near distribution boundaries may benefit from world‐model/predictive‐coding style objectives (e.g., JEPA, which learns representations by predicting missing or future parts of an observation in a shared embedding space) and from neurosymbolic or graph‐reasoning approaches that enable executable constraints and compositional generalization [135, 136, 137].

Despite these advances, key challenges remain. For inverse design, the central issue is how to embed complex, system‐level sustainability constraints directly into latent spaces. Current property‐conditioned methods (e.g., MatterGen [96], Con‐CDVAE [132]) can bias outputs toward single objectives but typically revert to post‐hoc filtering or reinforcement learning with sparse rewards under non‐differentiable rules. Addressing this requires architectures that integrate differentiable physics, process‐aware priors, and multi‐objective optimization so that sustainability drives generation intrinsically. For forward modeling, the critical challenge is ensuring physical fidelity: generated spectra or microstructures must remain consistent with thermodynamics and kinetics, not just statistical similarity [138]. Embedding physics‐based priors, leveraging experimental feedback, and validating in closed‐loop autonomous labs such as the A‐Lab [2] will be essential to align novelty with genuine sustainability.

### 2.3 Large Language Models for Materials Discovery and Knowledge Synthesis

Large Language Models (LLMs) are transforming materials discovery, evolving from passive text processors to active agents in knowledge synthesis and task‐oriented design [128, 139, 140, 141, 142, 143, 144, 145, 146]. A primary near‐term application is large‐scale knowledge extraction; domain‐specific models like MatSciBERT and MatBERT significantly outperform general LLMs in mining material properties, synthesis parameters, and latent relationships from literature to construct structured, machine‐readable databases [47, 83, 147, 148, 149, 150, 151, 152]. Beyond natural language, LLMs effectively process symbolic chemical and structural representations. Transformer models leverage string‐based formats like SMILES and SELFIES (e.g., ChemBERTa) for molecular property prediction and generation [153, 154, 155]. Similarly, models such as CrystaLLM utilize tokenized structural files (e.g., CIFs) to generate physically plausible crystalline materials [156]. Consequently, LLMs now seamlessly bridge textual knowledge extraction and the generative modeling of atomic structures.

In practice, reliable performance on materials tasks often benefits from domain adaptation of general‐purpose LLMs [157]. Parameter‐efficient adaptation and expert routing provide a practical route to inject domain skills while retaining general capabilities; for example, X‐LoRA dynamically composes low‐rank adapter experts via token‐level gating [158]. Complementarily, continued pretraining and supervised fine‐tuning, have been explored in materials‐oriented settings, highlighting trade‐offs between compute cost, domain coverage, and downstream performance [157]. Domain‐specific exemplars such as BioinspiredLLM illustrate how a domain‐adapted, fine‐tuned LLM can support materials–mechanics knowledge tasks and broader scientific assistance [159].

Another line is to augment LLMs with external knowledge and tools. Because sustainability constraints and synthesis knowledge are distributed across heterogeneous sources (e.g., papers, databases, facility documentation, and local experimental records), retrieval‐augmented and tool‐augmented LLMs offer a practical pathway to grounded, citation‐linked reasoning beyond static parametric knowledge [100, 160]. Such augmentation can also introduce failure modes (e.g., retrieval bias, incomplete corpora, and tool‐chain brittleness), motivating domain‐specific evaluation (including tool‐use benchmarks) and careful provenance reporting [161].

Building on such retrieval / tool augmentation, large language models (LLMs) are increasingly embedded in multi‐modal and agentic frameworks, where they serve as planning and reasoning hubs for multi‐step scientific workflows [162, 163, 164, 165]. For instance, a researcher might query: *“Suggest a bio‐based polymer with high tensile strength and optical transparency for food packaging.”* Recent frameworks in materials discovery, such as HoneyComb [166] and AtomAgents [97], instantiate LLMs as central planners that coordinate external tools, including databases, simulators and structure predictors, to accomplish multi‐step design tasks and illustrate how tool orchestration extends beyond single‐turn prompting [97, 165, 167]. Beyond monolithic model scaling, recent materials‐domain systems suggest that compositional, cross‐domain reasoning can be operationalized through explicit knowledge‐graph traversal and a division of labor across agents. For instance, GraphAgents [168] builds two complementary knowledge graphs from scientific corpora: a domain‐focused graph that supports deeper, in‐context reasoning, and a broader materials–properties graph that helps surface connections across subfields. It then assigns different agents to decompose the design question, retrieve supporting evidence, extract key requirements, and traverse the graphs to propose candidate solutions. The framework is illustrated on a sustainability‐motivated substitution task: identifying PFAS‐free (i.e., not relying on per‐ and polyfluoroalkyl substances, which are persistent fluorinated chemicals) candidates for biomedical tubing under friction, thermo‐chemical stability, and biocompatibility constraints. Related systems such as SciAgents [169], similarly use ontological knowledge graphs and role‐specialized agents to iteratively propose hypotheses for bioinspired materials, and should be paired with downstream verification (e.g., simulation or targeted experiments).

**FIGURE 6:**
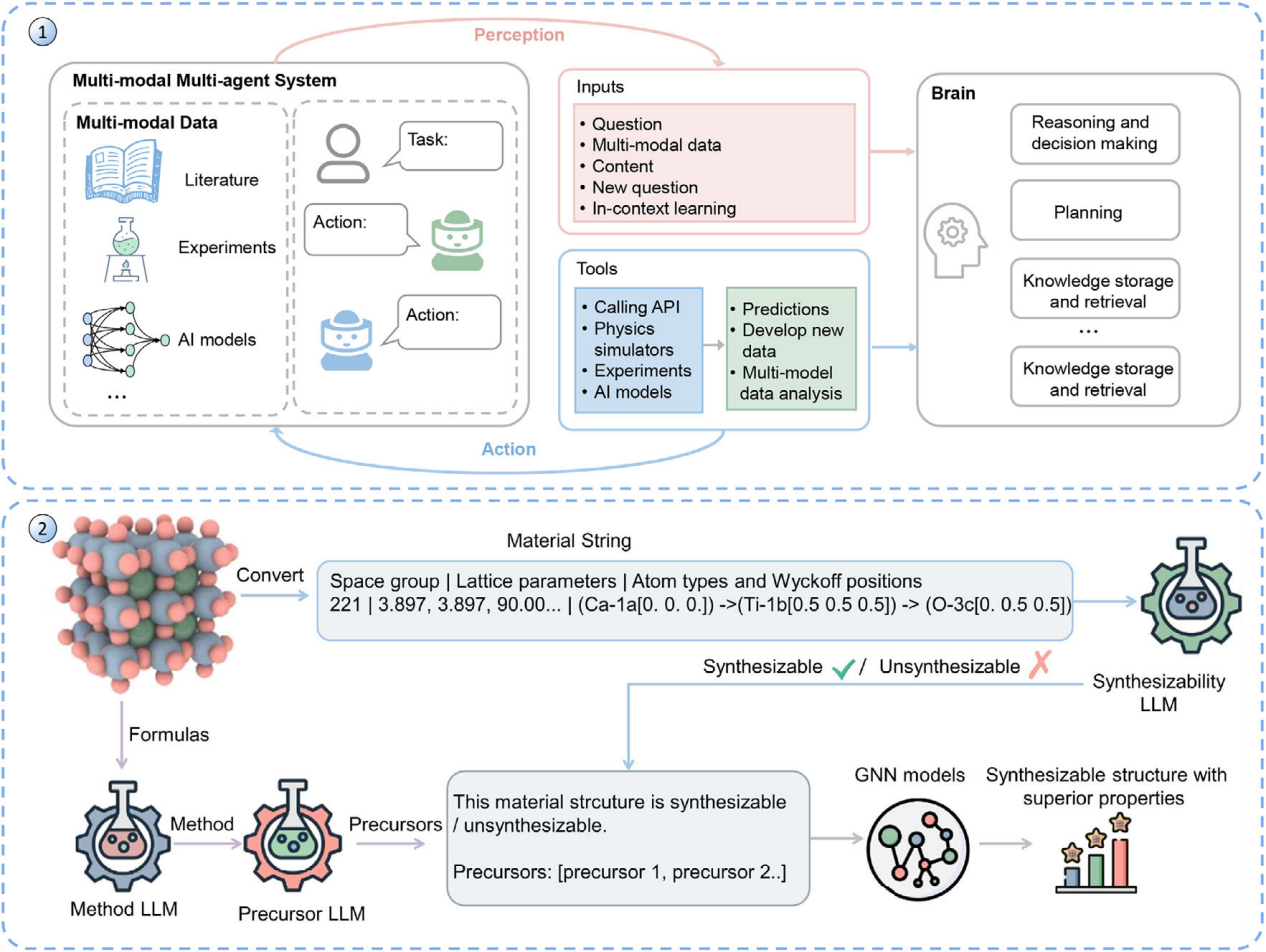
LLM‐orchestrated frameworks for materials design. (1) AtomAgents multi‐model, multi‐agent system, where a large language model coordinates specialized agents and external tools (databases, simulations, ML models) for automated alloy design and analysis [97]. (2) Crystal Synthesis LLM (CSLLM) framework, which encodes crystal structures as “material strings” and uses dedicated LLMs together with GNNs to predict synthesizability, synthesis routes, precursors, and key properties. Adapted from ref. [99], CC BY 4.0.

Benchmark suites such as MatTools aim to quantify reliability, tool‐use accuracy, and reasoning performance of LLMs on domain‐specific tasks [161]. Figure 6 illustrates representative realizations of this paradigm: Panel (1) depicts AtomAgents [97], a physics‐aware multi‐agent system in which an LLM coordinates specialized agents for literature retrieval, data analysis, code generation, and atomistic simulation, enabling automated multi‐step alloy design with minimal human intervention. Panel (2) presents the Crystal Synthesis Large Language Models (CSLLM) framework [99], which encodes crystal structures into concise material strings and employs dedicated LLMs, followed by graph neural networks, to infer synthesizability, synthesis routes, precursors, and key properties. Together, these examples illustrate how LLMs can orchestrate tools and data sources within end‐to‐end, closed‐loop materials design workflows.

A key motivation for agent architectures in materials discovery is improved robustness under distribution shift. Rather than relying on a single closed forward pass, agentic systems implement an iterative *propose–verify* loop, where an LLM proposes candidates or hypotheses and then uses external tools to gather evidence and perform tool‐grounded checks (e.g., retrieval from literature / databases, code execution, simulators, or surrogate models), revising decisions based on feedback. Here, “verify” refers to practical validation via evidence and consistency checks rather than formal guarantees. AtomAgents provides a representative realization of this paradigm by coupling LLM planning with specialized agents and physics‐aware tools for retrieval, analysis, and simulation [97].

Despite this progress, deploying LLMs and foundation models in materials discovery requires careful control of reliability. Documented failure modes include hallucinated facts, error amplification from biased or incomplete corpora, and missing or violated physical constraints [170, 171]. In practice, these risks are mitigated most effectively within a tool‐grounded, verification‐centric workflow: model outputs are grounded in external evidence through retrieval and explicit tool use (e.g., curated literature / databases), constrained by executable domain rules (e.g., stoichiometry, charge balance, and thermodynamic plausibility), and accompanied by uncertainty‐aware triage so that low‐confidence suggestions are prioritized for simulation or targeted experimental review [162, 172]. While elements of such grounding, constraint integration, and uncertainty awareness are beginning to appear in emerging agentic and autonomous platforms, systematic, domain‐specific evaluation of tool‐use reliability and end‐to‐end decision quality remains an open challenge [161].

To provide a structured overview beyond the narrative discussion, we summarize representative LLM‐centric paradigms for sustainable materials design in Appendix Table A1. The table consolidates each method family, its supported modalities, sustainability role, what it enables in practice, and key limitations.

### 2.4 Multi‐Modal AI‐Driven Optimization

Multi‐modal AI‐driven optimization enables targeted search in high‐dimensional materials design spaces under limited evaluation budgets. In sustainability‐oriented settings, the objective is typically multi‐objective: performance must be balanced against cost, resource criticality, manufacturability, and life‐cycle impact, often under hard feasibility constraints (e.g., phase stability, process windows, or safety limits).

Surrogate models make such optimization practical by approximating expensive evaluators (e.g., DFT, CALPHAD, or experiments) and supporting uncertainty‐aware decision making. Multi‐fidelity learning further improves efficiency by combining cheap but informative low‐fidelity signals with sparse high‐fidelity labels, so that most candidates can be triaged inexpensively while reserving high‐fidelity verification for a small set; reliable gains depend on cross‐fidelity correlation, calibration, and uncertainty‐aware validation [90, 91].

**FIGURE 7:**
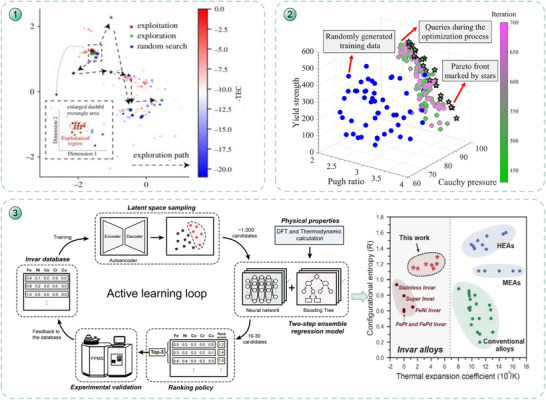
Examples of AI‐driven optimization in materials design. (1) Single‐objective active learning for sustainable alloy design (Invar case study): candidate alloys are sampled in a learned latent space using exploitation, exploration, and random search (color indicates thermal expansion coefficient, TEC). Reprinted from [173], CC BY 4.0. (2) Constrained multi‐objective Bayesian optimization in Mo‐Nb‐Ti‐V‐W: blue points denote the initial training set, colored points are the sequential queries (iteration‐coded), and stars mark the estimated Pareto front in objective space (Pugh ratio, Cauchy pressure, yield strength). Reprinted from [174], CC BY 4.0. (3) Multi‐modal active‐learning framework for Invar‐type HEAs [103]. Left: Generative–active workflow where candidates are proposed from a learned latent space and refined in an active‐learning loop with surrogates, CALPHAD / DFT screening and experiments. Right: Design space of configurational entropy versus thermal expansion, where discovered HEAs extend into a new low‐expansion, high‐entropy region.

A useful view of optimization pipelines is along three coupled aspects: objectives, models, and interaction. On the objective side, Figure 7 highlights both regimes: panel (1) illustrates single‐objective active learning (exploration–exploitation), while panel (2) shows constrained multi‐objective BO/ AL targeting an estimated Pareto front; evolutionary algorithms remain practical when diverse trade‐off solutions are desired [101, 102, 175]. On the model side, GP surrogates are common on moderate datasets, while higher‐dimensional settings often rely on learned representations for proposal generation coupled with surrogate / physics screening to maintain fidelity [176, 177]. On the interaction side, closed‐loop design‐evaluate‐update workflows selectively allocate high‐fidelity evaluations and can be organized as nested loops to improve sample efficiency (Figure 7, panel 3) [103, 178].

As these workflows become more sequential and long‐horizon, they motivate policy learning. The next subsection discusses reinforcement learning for optimizing such multi‐step decisions under competing objectives and constraints [105, 179].

### 2.5 Reinforcement Learning for Materials Discovery

**FIGURE 8:**
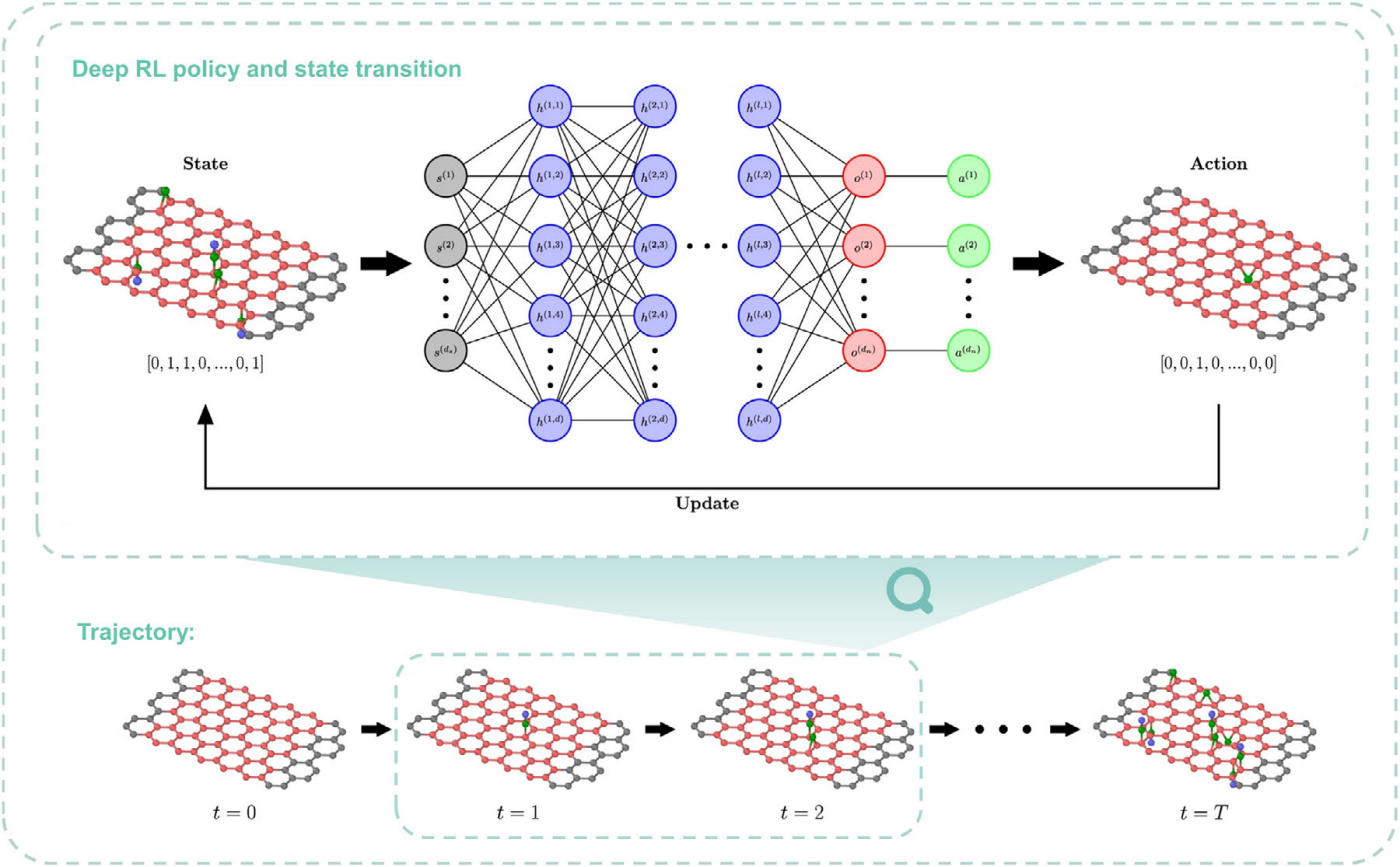
Reinforcement learning workflow for materials design. Schematic adapted from Zheng et al.: an RL agent observes an encoded atomic configuration (state), proposes a structural modification through a policy network (action), evaluates the modified structure via molecular dynamics to obtain a property‐based reward (e.g., toughness), and updates its policy to generate progressively improved designs. Adapted from ref. [104], CC BY 4.0.

Reinforcement learning (RL) provides a framework for sequential decision making under uncertainty [180]. In materials discovery, many design and synthesis problems can be cast as Markov decision processes, where the agent iteratively modifies a material or process and receives rewards computed from simulation or measurements (e.g., target performance, process feasibility, or resource / energy penalties) [104, 105, 106, 181, 182]. Figure 8 illustrates this general workflow.

Representative studies have demonstrated that RL can efficiently explore large design spaces and learn non‐trivial design policies. Zheng et al. [104] used deep RL to optimize graphene oxide by sequentially editing oxygen functional groups, achieving configurations with toughness exceeding almost all random baselines. Rajak et al. [183] applied RL to generate kirigami patterns in monolayer ${\mathrm{MoS}}_{2}$, discovering geometries with enhanced stretchability. Later, Karpovich et al. [105] applied RL to inverse inorganic materials design, where a deep RL agent proposed chemically valid compositions evaluated by surrogate models for formation energy and elastic properties. RL has also been coupled to process and experimental optimization. For example, Volk et al. [106] integrated RL with the AlphaFlow self‐driving microfluidic platform for closed‐loop multistep synthesis, and Park et al. [182] used deep RL to inversely design MOFs for ${\mathrm{CO}}_{2}$ direct air capture, providing a concrete example of RL‐driven design toward a climate‐relevant objective.

Within the broader multi‐modal AI landscape, RL can serve as a sequential decision layer that coordinates information from heterogeneous models and data sources. In such a framework, state representations may include not only compositional and structural descriptors, but also features derived from physics‐based surrogates or data‐driven models. Existing demonstrations show that RL can operate under complex, engineered reward functions and constraints [104, 105, 106]. As sustainability‐related metrics, such as embodied energy, elemental criticality, recyclability, and toxicity, become increasingly standardized and integrated into open datasets [184], these RL formulations could, in principle, be extended to co‐optimize performance and sustainability objectives within closed‐loop discovery and manufacturing systems.

## 3 Multi‐Modal AI‐Driven Sustainable Alloy Discovery

The integration of multi‐modal AI approaches has revolutionized sustainable alloy discovery by accelerating design cycles, predicting complex sustainability metrics, and enabling large language model (LLM)‐driven innovation. These advances allow materials scientists to navigate high‐dimensional design spaces while simultaneously optimizing environmental impact, economic viability, and performance characteristics.

### 3.1 Accelerating the Design of Eco‐Friendly Alloys

**FIGURE 9:**
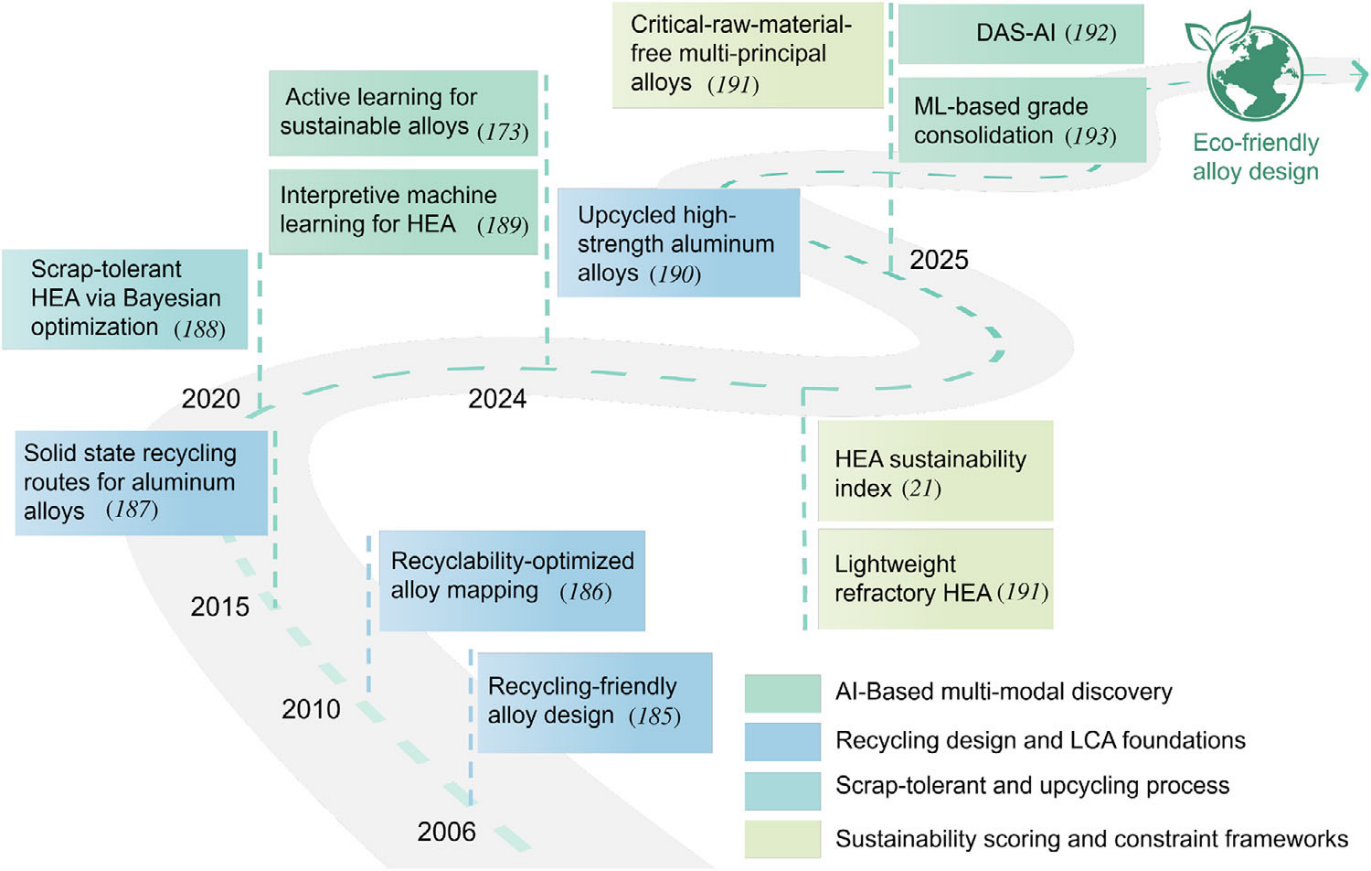
Representative advances in sustainable alloy design and AI‐driven optimisation. This roadmap highlights major methodological milestones toward sustainable alloy discovery. Early contributions focused on recycling‐aware alloy design and life cycle assessment. Later, advances include AI‐driven strategies such as scrap‐tolerant high‐entropy alloys (HEAs), solid‐phase upcycling of aluminum, machine learning–based grade consolidation, and multi‐objective optimization for CRM‐free and corrosion‐resistant alloy design. These efforts progressively integrate recyclability, criticality, and carbon impact considerations into the materials discovery pipeline.

**FIGURE 10:**
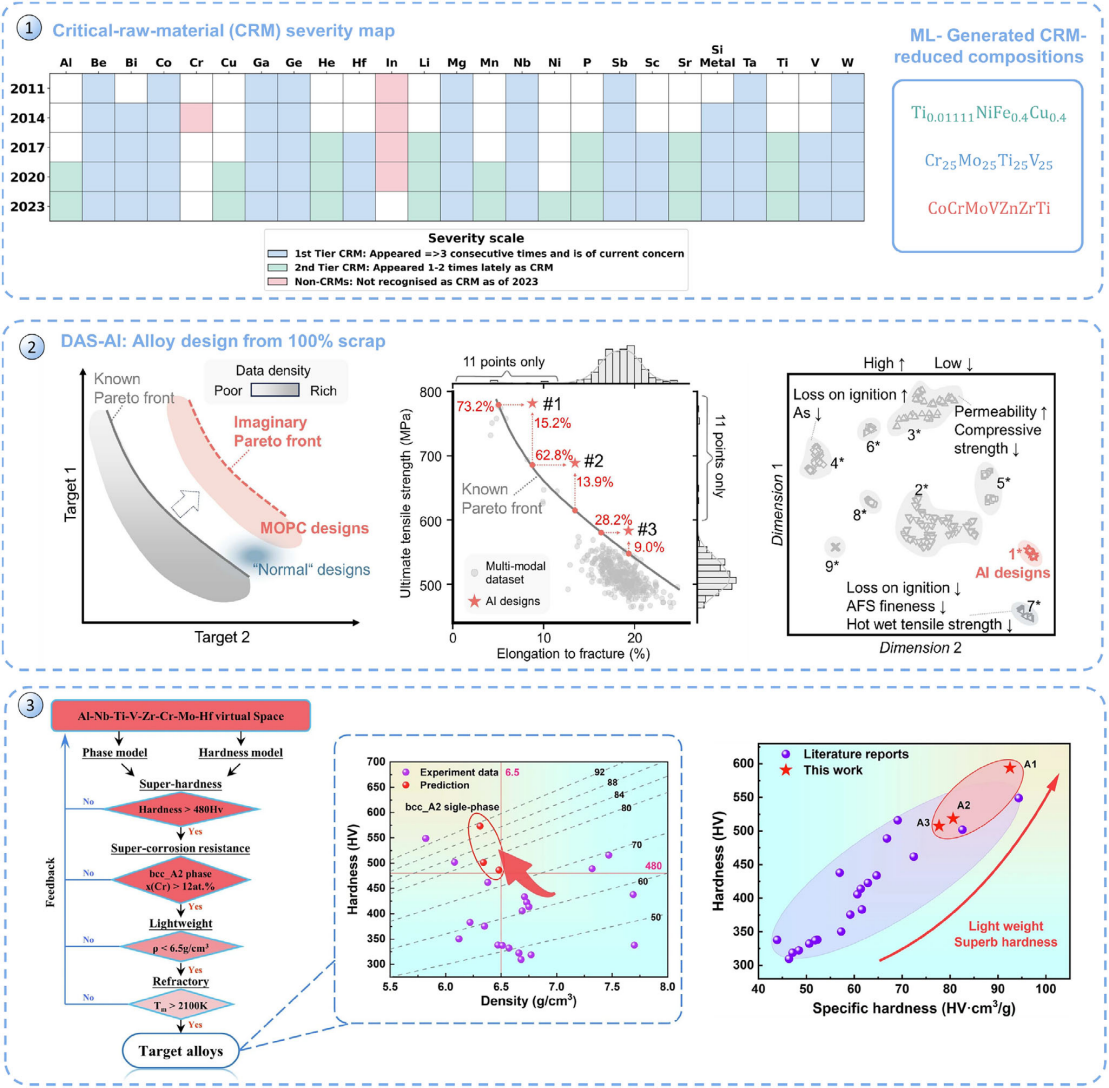
(1) Critical‐raw‐material (CRM) severity map motivating reduced‐CRM alloy design. ML methods contribute to CRM‐reduced composition designs. Reprinted from ref. [194], CC BY 4.0. (2) DAS‐AI: Design Alloys from the Scraps using multi‐modal AI. **Left**: A multi‐objective search uses an imaginary Pareto front to guide exploration into data‐sparse regions, balancing tensile strength and ductility. **Middle**: Predicted and experimentally measured strength–elongation results show obvious improvements in elongation and in strength compared with expert‐designed alloys. **Right**: A low‐dimensional projection of composition and processing descriptors shows that the AI‐proposed alloys form a distinct cluster from human‐designed datasets, indicating discovery beyond human bias. Reprinted from ref. [192], CC BY 4.0. (3) ML‐driven multi‐objective design and validation of lightweight refractory high‐entropy alloys (LW‐RHEAs). Reprinted from ref. [191], CC BY 4.0.

Eco‐friendly alloy design aims to deliver mechanical performance while lowering life‐cycle carbon footprints, enhancing recyclability, and reducing dependence on critical raw materials (CRMs) (as shown in Figure 10 panel 1) or heavy refractory elements [187, 194]. Recent sustainability frameworks for high‐entropy and multi‐principal element alloys formalize these objectives using composite indices that combine ${\mathrm{CO}}_{2}$ footprint, supply‐risk metrics, and production ratios, and then score tens of thousands of candidate HEAs to identify sustainability‐favorable composition families [21]. A roadmap highlighting the major methodological milestones toward sustainable alloy discovery is summarized in Figure 9.

**Lower‐Carbon Pathways via Recycling and Upcycling**. Life‐cycle assessments show that increased use of recycled metal significantly lowers overall energy use and emissions [185, 187]. Design‐for‐recycling strategies can further boost recyclability by adjusting alloy compositions to tolerate impurities and mixing of scrap streams without degrading mechanical properties [186]. Building on this principle, Wang et al. developed a solid‐phase upcycling route that converts 6063 Al scrap with minor copper, zinc, and magnesium additions into a 7075‐like high‐strength alloy in a single solid‐phase step. The upcycled alloy enhances both yield and ultimate tensile strength while maintaining good ductility [190].

**AI to Improve Recyclability and Tolerate Scrap Variability**. From a circular‐economy perspective, the proliferation of commercial alloy grades complicates scrap sorting and leads to downgraded secondary products [186]. Tiwari et al. addressed this challenge in 6xxx Al by applying PCA and k‐means clustering to 292 industrial data sets spanning 42 grades and multiple tempers, ultimately identifying ten optimal grades that preserve mechanical, corrosion, and technological performance while simplifying the design space, facilitating more robust scrap consolidation [193]. At the conceptual level, Barnett et al. introduced a scrap‐tolerant HEA design strategy by using Bayesian optimisation over Fe–Ni–Cr‐based systems to discover alloys whose strengths remain within targeted windows even when feedstock compositions vary, as would occur in practical scenarios involving mixed end‐of‐life Ni alloys and stainless steels, demonstrating the feasibility of designing alloys robust to compositional fluctuations in recycled inputs [188]. Complementing these efforts, Yang et al. proposed the DAS‐AI pipeline, which designs ductile iron from 100% scrap by learning from a multi‐modal dataset (composition, processing parameters, and microstructural descriptors) and coupling convolutional‐neural‐network surrogates with a tree‐based optimisation algorithm. DAS‐AI identifies alloys that use high‐impurity recycled feedstocks yet achieve higher elongation and tensile strength (illustrated in Figure 10 panel 2), while reducing manufacturing cost [192]

**AI‐Guided Trade‐Offs Between Mechanical Performance and Sustainability**. Machine‐learning‐based multi‐objective optimisation enables explicit trade‐offs between mechanical performance and sustainability metrics such as CRM content, density, or dependence on heavy refractory elements [21, 194]. Gao et al. developed a data‐driven workflow for lightweight refractory HEAs (LW‐RHEAs), integrating ML models with multi‐objective screening to identify candidates satisfying constraints on phase stability, density, melting point, hardness, and pitting potential [191]. In parallel, Singh et al. combined an Extra Trees Regressor (ETR) with metaheuristic optimization to identify multi‐principal element alloys with reduced critical raw material (CRM) content, achieving predicted hardness comparable to Nb‐ and Ta‐rich benchmarks while partially or fully substituting these high‐risk elements [194].

Taken together, these works outline a roadmap in which sustainability‐aware alloy design increasingly incorporates richer data modalities and explicit eco‐metrics. Current pipelines already combine composition descriptors, thermodynamic and phase features, corrosion data, and supply‐risk metrics within ML‐driven optimisation loops [21, 191, 193, 194], while DAS‐AI demonstrates that multi‐modal optimisation over composition, processing, and microstructure can directly exploit 100% scrap without sacrificing mechanical performance [192]. Extending these frameworks toward fully autonomous multi‐modal AI by incorporating process trajectories, microstructural images, life‐cycle assessment outputs, and textual knowledge on regulatory trends and CRM criticality, represents a promising direction for alloy design aligned with sustainability goals.

### 3.2 Predicting Key Sustainable Materials Properties (LCA, Criticality, Longevity, Corrosion)

**From Post Hoc Checks to in‐the‐Loop Prediction**. To design sustainable materials fundamentally relies on the ability to anticipate and quantify the properties that define sustainability. System‐level indicators, such as life‐cycle assessment (LCA), elemental criticality, and long‐term durability, are rarely direct outputs of first‐principles simulations, instead requiring the integration of heterogeneous, sparse, and indirect data sources. This necessitates a shift from post‐design assessment to predictive, in‐the‐loop modeling. Recent advances in multi‐modal AI provide a key enabler for this transition, by combining structured descriptors (e.g., composition, processing, microstructure) with unstructured or temporal data (e.g., images, degradation curves, and textual knowledge). Such cross‐modal architectures have shown promise in improving representation quality and the prediction of specific sustainability‐related metrics [76, 121, 192].

**From Post Hoc Checks to in‐the‐Loop Prediction**. To design sustainable materials fundamentally relies on the ability to anticipate and quantify the properties that define sustainability [51, 195]. However, rigorous frameworks like Life‐Cycle Assessment (LCA) involve complex inventory analyses and boundary definitions that are rarely direct outputs of first‐principles simulations [16, 196]. Instead of claiming full LCA integration, current approaches increasingly rely on environmental proxies—such as Global Warming Potential (GWP), embodied energy, or synthesis accessibility—as tractable optimization objectives [55]. This necessitates a shift from post‐design assessment to predictive, in‐the‐loop modeling guided by these key indicators [51]. Recent advances in multi‐modal AI provide a key enabler for this transition, by combining structured descriptors (e.g., composition, processing, microstructure) with unstructured or temporal data (e.g., images, degradation curves, and textual knowledge). Such cross‐modal architectures trained on these complementary views consistently improve both representation quality and the prediction of specific sustainability metrics [76, 121, 192].

**FIGURE 11:**
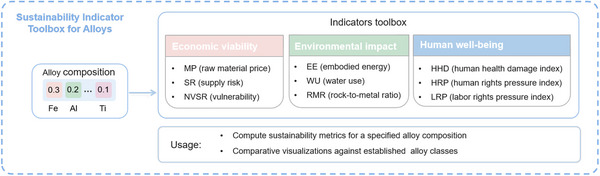
From alloy composition to a 9‐indicator sustainability vector (MP, SR, NVSR, EE, WU, RMR, HHD, HRP, LRP) for benchmarking and visualization.

**Composable Sustainability Indicators**. Critical‐raw‐material assessments are increasingly incorporated into materials‐selection frameworks for resource‐risk‐aware design [197, 198]. Building on these, *Alloy Sustainability* dataset (Figure 11) provides a reproducible workflow to compute a nine‐indicator sustainability vector directly from alloy composition (mass fractions), enabling comparative screening and benchmarking, and supporting downstream multi‐objective design studies alongside performance targets [184].

**Learning Degradation From Images and Time Series**. For sustainability‐aware alloy design, durability and corrosion must be inferred from limited, heterogeneous evidence. Image‐based models quantify localized corrosion features (e.g., small cavities formed by pitting corrosion) and surface‐film coverage from micrographs, enabling high‐throughput, reproducible morphology measurements [199, 200]. Complementarily, data‐driven forecasters trained on electrochemical time series extrapolate long‐term stability from short‐term exposure, supporting efficient screening of lifetime under realistic environments [201, 202]. When embedded in closed‐loop, active‐learning experimentation, these models prioritize the most informative measurements and steer composition–process choices toward candidates that jointly satisfy performance and sustainability objectives [111]. Evidence across advanced alloy classes further indicates systematic trade‐offs between functional properties and corrosion tolerance, underscoring the value of concurrent optimization in sustainability‐aware design [191].

**Forecasting Service Life: Mechanisms and Uncertainty**. Predicting material longevity requires mapping short‐term, accelerated observations to long‐term failure mechanisms—a task where AI has shown promise only when grounded in specific degradation physics. In the battery domain, data‐driven models have successfully predicted the remaining useful life (RUL) of lithium‐ion cells by extracting non‐linear features from early‐cycle voltage discharge curves, effectively correlating short‐term degradation signatures with long‐term capacity fade [203]. Similar approaches are being adapted for structural alloys and concrete, where computer vision and time‐series networks quantify localized damage evolution (e.g., pitting corrosion depth or chloride ingress) from accelerated aging tests [199, 200, 201]. However, purely data‐driven extrapolation carries inherent risks when applied to unseen service conditions. To mitigate this, recent frameworks increasingly incorporate *uncertainty quantification* (UQ) and physics‐informed constraints (e.g., PINNs) to ensure that lifetime predictions remain physically plausible outside the training domain [202, 204]. When embedded in closed‐loop experimentation, these probabilistic models allow algorithms to prioritize the most informative measurements, steering composition–process choices toward candidates that robustly satisfy both performance and durability objectives [111, 191].

### 3.3 Leveraging LLMs for Sustainable Alloy Design

Large language models (LLMs) are emerging as versatile tools for alloy innovation by linking textual materials knowledge, structured alloy data, and computational tools into unified workflows. Recent analyses argue that such models can help integrate knowledge across literature, databases, and simulations, and thus have potential to support more holistic and constraint‐aware design strategies, including resource and processing considerations [205, 206, 207].

LLM‐based models are beginning to capture alloy design relationships directly from language‐like representations. Chaudhari et al. introduced AlloyBERT, a transformer model which uses alloy descriptions that combine composition and processing information to predict elastic modulus and yield strength, and achieves more accurate results than conventional machine‐learning baselines, indicating that language‐based representations can capture useful metallurgical patterns [208]. Ni et al. developed AlloyGPT, an alloy‐specific generative language model that encodes composition, phase and property information in a unified textual format and supports both forward property prediction and inverse alloy design for additively manufacturable Al‐based alloys. The generated candidates satisfy targeted printability and strength criteria as verified against CALPHAD‐based simulations and prior experimental knowledge [209]. Beyond property prediction, LLMs are also being adapted for thermodynamic and phase reasoning [210], achieving more efficient interpretable exploration of composition spaces. These studies indicate that alloy‐focused language models can act as flexible surrogates that link textual domain knowledge with quantitative alloy property prediction and candidate generation [208, 209].

Recent efforts have also integrated LLMs into agent‐based and tool‐using design frameworks. The physics‐aware AtomAgents framework [97] uses an LLM planner to coordinate retrieval, uncertainty‐aware learning, and atomistic simulations, enabling automated screening of alloy candidates under performance and compositional constraints. Complementary systems such as HoneyComb [166] and MatAgent [211] use the similar paradigm, where LLMs act as orchestrators that call external tools for database queries, property evaluation, or structure generation rather than relying on free‐form text generation. Evaluation suites like MatTools [161] formalize benchmarks for tool use and reasoning reliability, providing a quantitative basis for assessing alloy‐oriented agents. More broadly, generative agent frameworks equipped with explicit APIs can propose chemically valid alloy compositions and refine them through formation‐energy and stability predictors [207].

In the context of sustainable alloy design, LLM‐driven frameworks provide a natural way to encode and enforce qualitative constraints. Queries such as“avoid elements with high supply risk” can be translated by LLM or agent systems into explicit filters or objectives applied to thermodynamic simulations, mechanical‐property predictors, or sustainability databases [184]. In this emerging paradigm, LLMs serve as interface and orchestration layers that align alloy discovery with sustainability‐aware objectives, while quantitative validation remains grounded in established CALPHAD, phase‐field, and mechanical modeling frameworks.

## 4 Recyclable and Reusable Materials

### 4.1 Designing Recyclable and Reusable Materials

**FIGURE 12:**
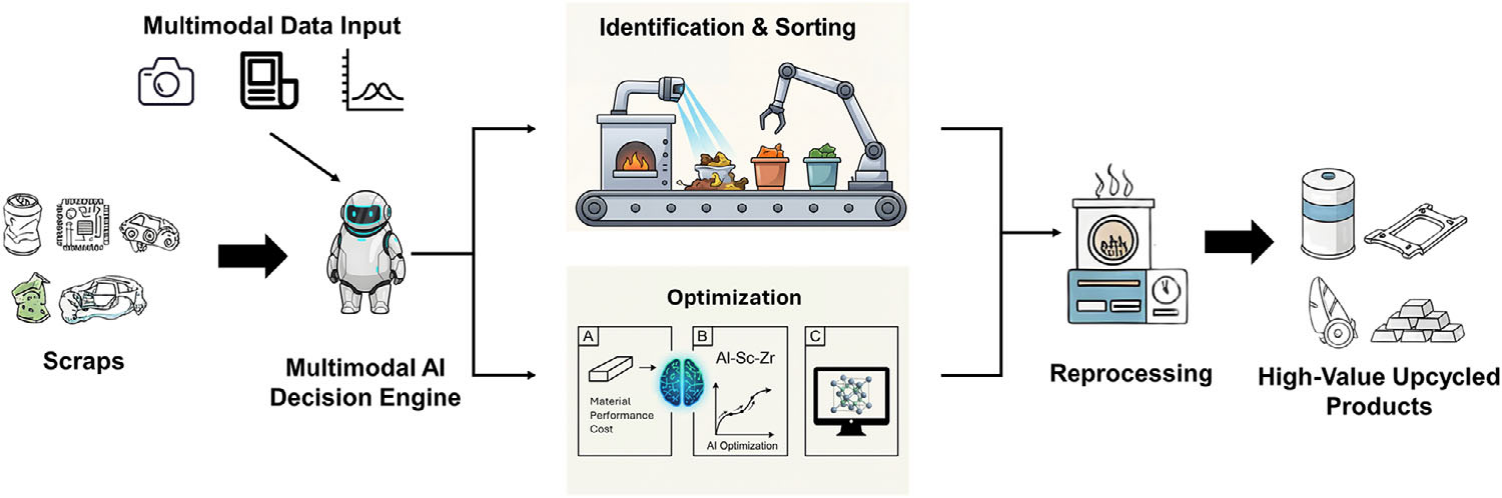
Overview: a schematic diagram of an integrated framework where multi‐modal AI enables the upcycling of scrap materials into high‐value products through automated identification, sorting, and reprocessing optimization.

Traditional material design methods have often relied on trial‐and‐error cycles to validate hypotheses, with notable drawbacks of inefficiency and high costs [212]. For recycled feedstocks and secondary alloys, heterogeneous waste sources introduce complex and variable impurity profiles, making composition control and property reproducibility more challenging and increasing the experimental burden of trial‐and‐error optimization [213]. Consequently, recycled metals and alloys are frequently downcycled, as contamination and compositional uncertainty can limit direct closed‐loop reuse in high‐performance applications [214]. As illustrated in Figure 12 AI‐assisted alloy design can improve research and development efficiency by jointly optimizing composition, processing, and other high‐dimensional factors, thereby reducing experimental trial‐and‐error, cost, and material/energy consumption [24, 50]. Such data‐driven workflows also enable multi‐objective optimization across multiple performance metrics [120]. In specific recycling‐upgrade studies, targeted alloying and process optimization have been reported to recover and, in some cases, improve key properties relative to common benchmark grades, providing practical routes for alloy up‐cycling [215].

**FIGURE 13:**
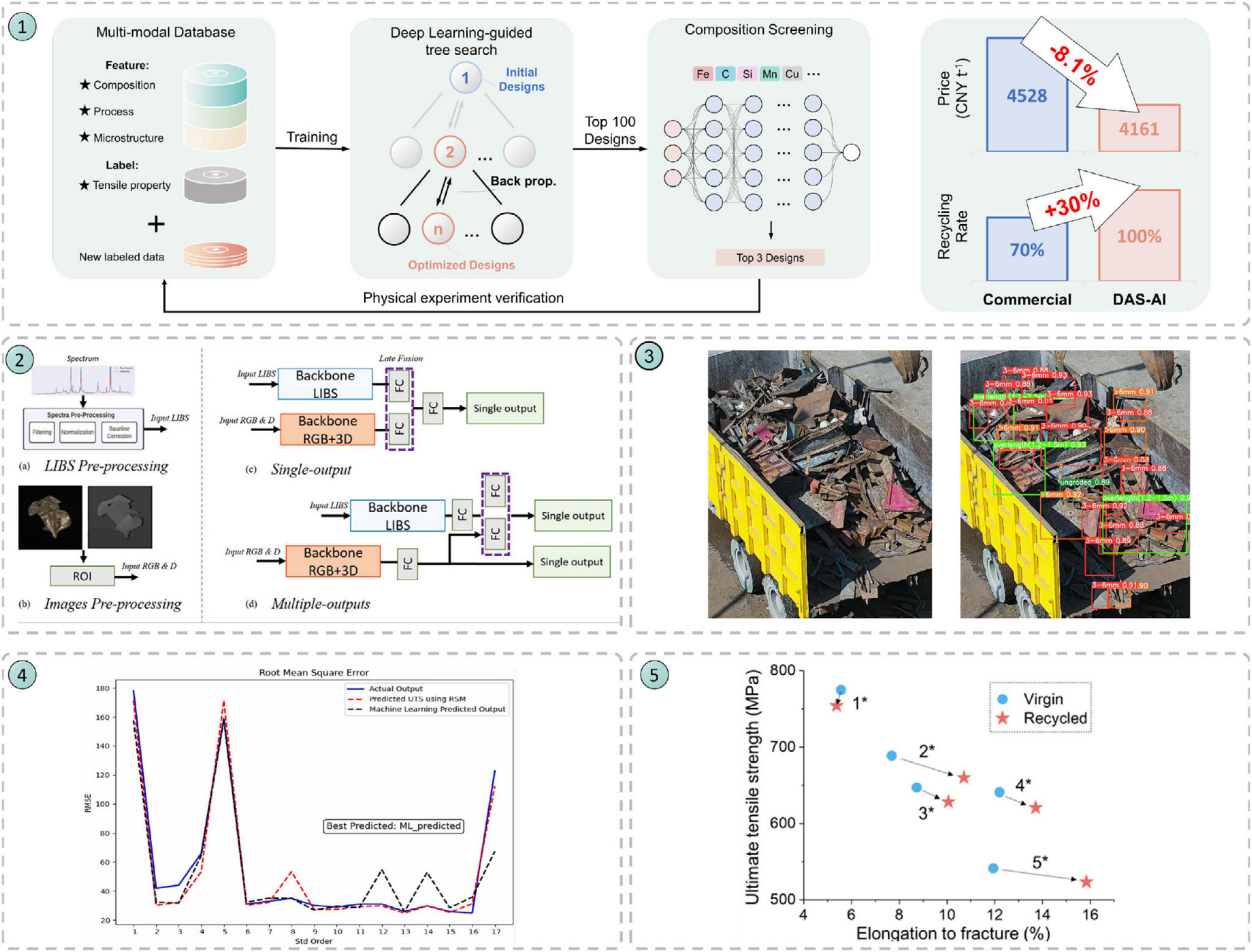
(1) The DAS‐AI overview and its multiple model and experimental performance. Adapted from ref. [192], CC BY 4.0. (2) Two new methods using deep learning models to fuse RGB and depth images with LIBS data, achieving more recycling of waste aluminum materials and reducing quality loss during the recycling process. Reprinted with permission from ref. [218], Elsevier. (3) The application of the CNIL‐Net scrap‐quality inspection model post‐inference, detailing each scrap item with its category label and confidence level. Reprinted with permission from ref. [219], CC BY 4.0. (4) Illustrates the performance evaluation of the ML model using RMSE. Reprinted with permission from ref. [220], CC BY 4.0 (5) The performance of DAS‐AI. Reprinted with permission from ref. [192], CC BY 4.0.

As shown in Figure 13 (panel 1), Wei et al. [192] conducted research on ductile iron recycling, using 100% scrap. Using a multi‐modal database and DAS‐AI models, they identified design principles for cast iron materials enabling compositional‐structural control and performance optimization. Results showed substantial gains in tensile strength and ductility, along with a noticeable reduction in cost, achieved through improved waste utilization and process optimization. AI can help design various materials, not limited to recycled ductile iron. Alireza Vahid et al. [216] used 7075 aluminum alloy scrap as the base material and added a certain amount of Sc and Zr to the scrap. They combined Bayesian optimization with an experimental optimization workflow to refine composition and heat‐treatment parameters, promoting precipitation strengthening through the formation of multiple strengthening precipitates [216]. Experimental results indicated improved strength relative to the baseline alloy condition studied, with ultimate tensile strength increases on the order of tens of MPa under optimized Sc and Zr additions and heat treatment [216]. Furthermore, as the number of iterative optimizations increased, the performance parameters of various alloys became more stable, demonstrating that this design approach combining Bayesian optimization and the AEO framework can operate reliably and consistently. Li et al. [215] explored upcycling of aluminum scrap via targeted microalloying additions, including Sc, to improve mechanical performance and microstructural quality. They compared several machine‐learning baselines, including ELM, BP neural networks, SVM, and RF, using a standardized pipeline of data curation, feature selection, model training, and property prediction [215]. The additive identity and its concentration were treated as explicit input variables in the predictive models [215]. Within the studied composition range, an Sc addition of 0.1% was identified as a favorable level, improving mechanical properties while refining microstructure and reducing defect density and size [215].

Furthermore, Torralba et al. [217] explored the feasibility of using multi‐component alloy mixtures from electronic waste as direct alloy feedstocks, reducing reliance on fully refined primary metals. Collectively, these studies suggest that AI‐assisted workflows can support composition and microstructure‐aware optimization in recycled‐alloy design. Such approaches can contribute to higher‐value recycling outcomes by improving alloy properties and reducing defect formation, and in some settings may increase effective closed‐loop recycling fractions. AI can also complement expert heuristics by integrating physics‐based descriptors with larger multi‐modal datasets, enabling more systematic exploration and optimization of alloy design spaces. Looking forward, large language model enabled tool‐use and planning may further strengthen evidence‐grounded design workflows, while advances in process modeling and control could expand practical routes for recovering complex alloy feedstocks from waste streams.

### 4.2 AI‐Enabled Material Recycling: Identification, Sorting, and Reprocessing Optimization

In material recycling, traditional manual sorting is inefficient and is often performed under unfavorable working conditions [221]. AI‐based sensing and decision models can improve efficiency and classification accuracy across key recycling stages, which can increase effective sorting quality and recycling yield [221, 222, 223]. However, waste‐identification performance varies across models and operating conditions, motivating comparative evaluation of reliability and accuracy under representative deployment scenarios [224, 225].As shown in Figure 13 (panel 2), Díaz‐Romero et al. [218] combined LIBS with RGB and depth imaging in deep‐learning fusion models, including single‐output and multiple‐output variants, to classify post‐consumer aluminum scrap into alloy groups. Experimental results indicated that the single‐output fusion model performed strongly for separating casting and wrought scrap, and that the multi‐output formulation achieved consistently high performance for the three‐fraction classification task (PREM, DEX, and SEC) [218].

Classification is a key step in material recycling because it groups waste streams with similar compositions or properties for subsequent processing [226, 227]. Without sufficiently detailed classification, downstream utilization becomes less reliable, and mixed streams may introduce composition variability or impurities that compromise recyclate quality and material performance [228, 229]. As shown in Figure 13 (panel 3), Xiao et al. [219] developed the CNIL‐Net model to address the challenge of inspecting dense and overlapping scrap in industrial settings. By integrating a small‐object detection layer, a Coordinate Attention mechanism, and Soft‐NMS into the YOLOv5 architecture, the model achieved an mAP of 96.5% on a nine‐category scrap dataset. Similarly, Xu et al. [230] established a steel‐scrap image dataset and developed the CSBFNet model for multi‐category scrap classification and rating. Their results showed an average accuracy of 92.4% for the full category, with an mAP of 90.7%, demonstrating the potential of deep‐learning‐based systems for intelligent scrap inspection and sorting.

Reprocessing of recycled alloys represents the final and most critical stage in the entire recycling process, aiming to further refine processed scrap materials into high‐quality recycled alloys [213]. Effective reprocessing techniques can significantly improve material performance and optimize material characteristics [231, 232]. As illustrated in Figure 13 (panel 4), Al‐Alimi et al. [220] studied the process optimization for the reprocessing of 6061 aluminum alloy scrap. Through forging reprocessing with reinforced (RSM), they established fitting formulas. Experimental batches revealed optimal particle addition ratios within specific ranges and relatively suitable processing conditions, therefore improving UTS and hardness metrics in scrap reprocessing.

In summary, identifying and classifying alloy scrap facilitates centralized processing of materials with similar compositions and properties, enabling efficient high‐quality reprocessing and better material upgrading and optimization.

## 5 Sustainable Autonomous Laboratory

**FIGURE 14:**
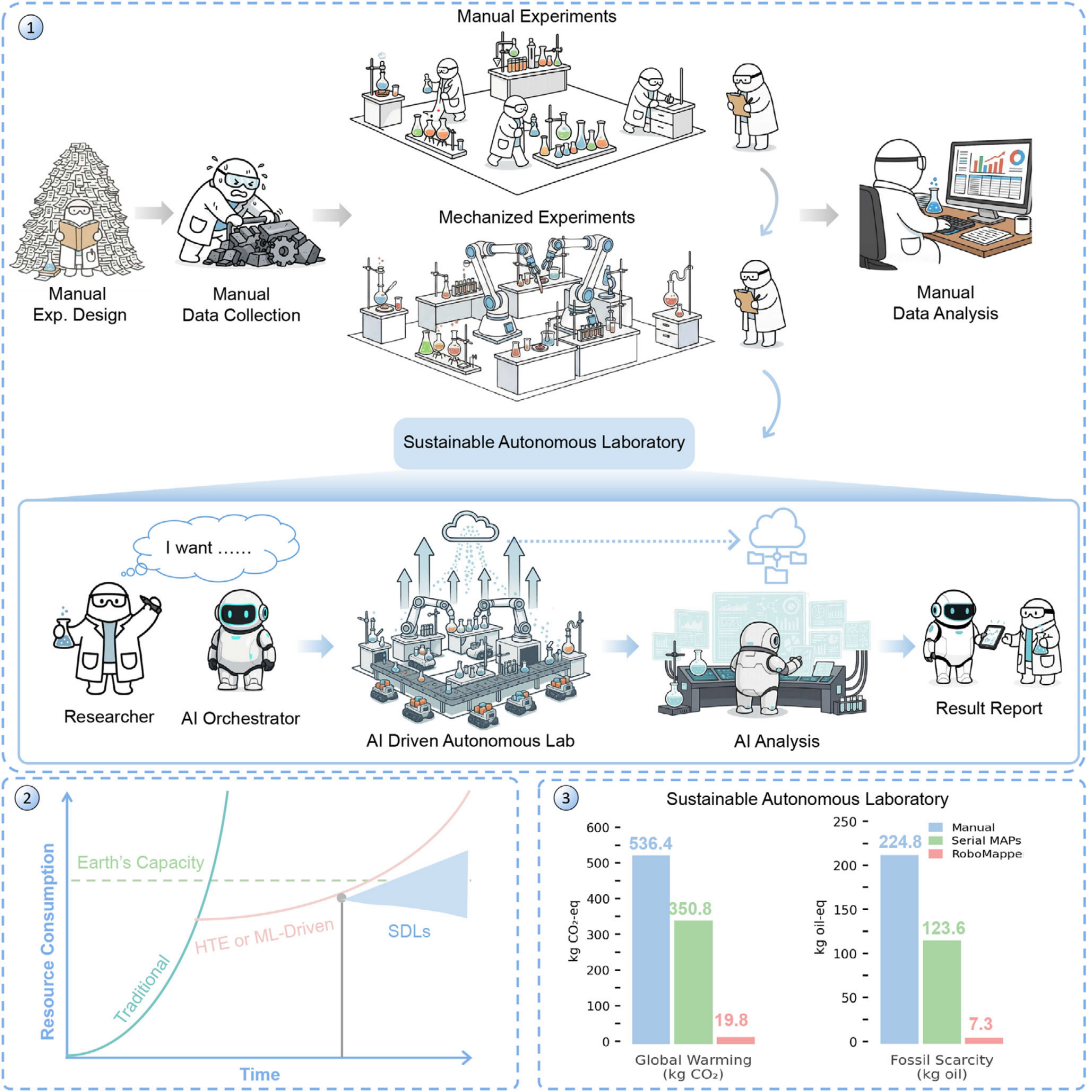
From automation to sustainable autonomy. (1) Evolution from manual and mechanized experimentation to AI‐driven autonomous laboratories integrating multi‐model analysis. (2) Conceptual framework illustrating the convergence of automation, robotics, and AI toward self‐driving laboratories that can accelerate discovery while reducing material and energy consumption [233]. (3) Sustainable materials acceleration platform “RoboMapper” shows an order‐of‐magnitude decrease in environmental impacts [234].

Autonomous laboratories extend multi‐modal AI into physical experimentation by closing the design‐make‐test‐learn cycle with robotic execution and algorithmic decision‐making [2, 69]. In the context of sustainable materials design, this paradigm serves as a dual role: it enables the discovery process to proceed more frugally, with fewer samples, less waste, and shared infrastructure [233, 235], and it provides a programmable scientific agent where material objectives such as recyclability, elemental criticality, durability, and benign chemistry can be optimized alongside performance [236, 237]. Public discussions, including a 2025 *Nature* feature asking whether AI systems could ever merit a Nobel Prize, have highlighted the broader shift toward algorithmic co‐discovery [238]. As illustrated in Figure 14, the experimental paradigm is evolving from manual work and mechanized automation toward algorithmic co‐discovery, in which AI orchestrators bridge human intent and machine execution.

**Digital twins vs. self‐driving laboratories (definition and criteria)**. Although the term “digital twin” is sometimes used loosely, we adopt an operational definition that emphasizes dynamic synchronization: a digital twin couples a computational representation with a physical counterpart and is dynamically updated through bidirectional data flows as conditions change, supporting prediction and decision‐making [239, 240]. By contrast, many platforms discussed here perform closed‐loop experiment selection (e.g., Bayesian optimization or active learning) without demonstrating persistent bidirectional synchronization of the underlying physical asset; we therefore refer to these systems as self‐driving laboratories (SDLs) or closed‐loop experimentation unless such synchronization is explicitly shown.

### 5.1 From Automation to Autonomy

Early self‐driving laboratories demonstrated that automation coupled with machine learning could drastically accelerate experimental optimization. The “Ada” platform autonomously mapped the Pareto front between processing (annealing) temperature and film conductivity for combustion‐synthesized palladium films, using multi‐objective optimization to explore trade‐offs using an a posteriori multi‐objective optimizer (qEHVI), avoiding pre‐specified weighting of objectives [241]. The solid‐state “A‐Lab” further advanced this concept by integrating ab initio phase‐stability data, synthesis heuristics learned from text‐mined literature, ML‐driven XRD interpretation (confirmed with automated Rietveld refinement), and active learning to plan and execute solid‐state syntheses. Over 17 days of continuous operation, it synthesized 36 of 57 target materials; analysis of failed syntheses provided direct and actionable guidance on screening and synthesis‐design failure modes [2]. Complementary efforts in chemistry have demonstrated closed‐loop platforms such as “Clio,” which coupled robotic liquid handling with Bayesian optimization to identify fast‐charging lithium‐ion battery electrolytes six times faster than random search [242], and mobile‐robot systems that autonomously shuttle samples between synthesis and characterization stations while sharing existing instruments with human researchers [107].

Together, these platforms mark a shift from high‐throughput automation to adaptive, knowledge‐integrating autonomy: they use prior data to avoid redundant experiments, draw on diverse measurements to suppress false positives and downstream rework, and rely on modular, shared infrastructure rather than bespoke setups. Taken together, these characteristics naturally support more resource‐efficient and reproducible materials discovery workflows, and they provide a practical basis for treating sustainability as an explicit objective in autonomous laboratory design.

### 5.2 Toward Sustainable Autonomy

Autonomous laboratories provide an opportunity to couple accelerated discovery with more efficient use of materials, energy, and infrastructure. The solid‐state “A‐Lab” illustrates how multi‐modal information, including computational stability data, synthesis rules mined from the literature, and diffraction‐based feedback, can be integrated to plan informative experiments and reuse knowledge from failures, which helps reduce repeated exploration of unpromising candidates [2]. Autonomous mobile‐robot systems show that robotic platforms can operate in shared laboratory environments and interface with existing instruments, suggesting a path toward more widely deployable and less resource‐intensive autonomous infrastructures [107]. Recent perspectives explicitly connect such architectures to sustainability and accessibility, arguing that self‐driving laboratories, if designed with resource use in mind, can reduce the environmental and labor footprint of research and support wider, more equitable deployment of advanced experimentation. [233, 243, 244].

Multi‐modal AI within self‐driving laboratory frameworks provides a route to process‐level decision making in materials discovery and, in principle, allows sustainability considerations to be incorporated as explicit objectives or constraints when measurable [233, 245]. Studies of autonomous optimization in thin films and electrolytes demonstrate that Bayesian optimization and related strategies can efficiently navigate multi‐parameter spaces and map trade‐offs between competing objectives, for example electrical performance versus processing temperature or formulation complexity [241, 242]. Building on this, Volk and Abolhasani propose quantitative metrics for self‐driving labs, including throughput, optimization efficiency, material usage, and reproducibility, to benchmark how effectively different platforms use experimental resources to achieve their objectives [246]. Together, these works suggest that multi‐modal AI in self‐driving laboratories can, in principle, support process‐level decision making in which sustainability‐related quantities, such as resource consumption or critical‐element use, are incorporated as explicit objectives or constraints alongside conventional performance metrics, provided that these quantities are well defined and measurable [241, 242, 245, 246].

Closed‐loop design, synthesis, and characterization coupled to sustainability considerations is beginning to appear in practical platforms. The “RoboMapper” sustainable materials acceleration platform miniaturizes composition libraries onto palletized thin‐film chips, integrates high‐throughput optical and stability measurements, and embeds life‐cycle assessment within the same workflow [234]. Wang et al. report an LCA‐based reduction on the order of ten‐fold for generating perovskite stability data, while identifying stable wide‐bandgap metal‐halide perovskite alloys [234]. These results, viewed together with autonomous platforms such as Ada, A‐Lab, and closed‐loop electrolyte optimization [2, 241, 242], outline a concrete template for sustainable autonomy: multi‐modal AI reduces uninformative trials, autonomous workflows leverage shared and modular infrastructure, and selected platforms explicitly quantify the environmental impacts of discovery campaigns.

## 6 AI‐Designed Materials for Energy Transition and Carbon Capture

Energy‐storage, catalysis, and separation materials often face accelerated aging and performance decay under realistic operating conditions (e.g., cycling, humidity, reactive atmospheres, and temperature fluctuations). Compared with conventional structural materials, their optimization typically involves multiple coupled objectives beyond mechanical integrity, such as electrochemical kinetics, ionic transport, and adsorption–desorption performance, which makes purely manual trial‐and‐error development slow and resource‐intensive.

### 6.1 Advanced Materials for Energy Storage and Conversion

For batteries and catalysts, sustainability‐relevant challenges include critical raw material usage and energy‐intensive manufacturing. Data‐driven modeling can reduce experimental trial‐and‐error by prioritizing promising chemistries and operating windows before costly synthesis and testing.

**FIGURE 15:**
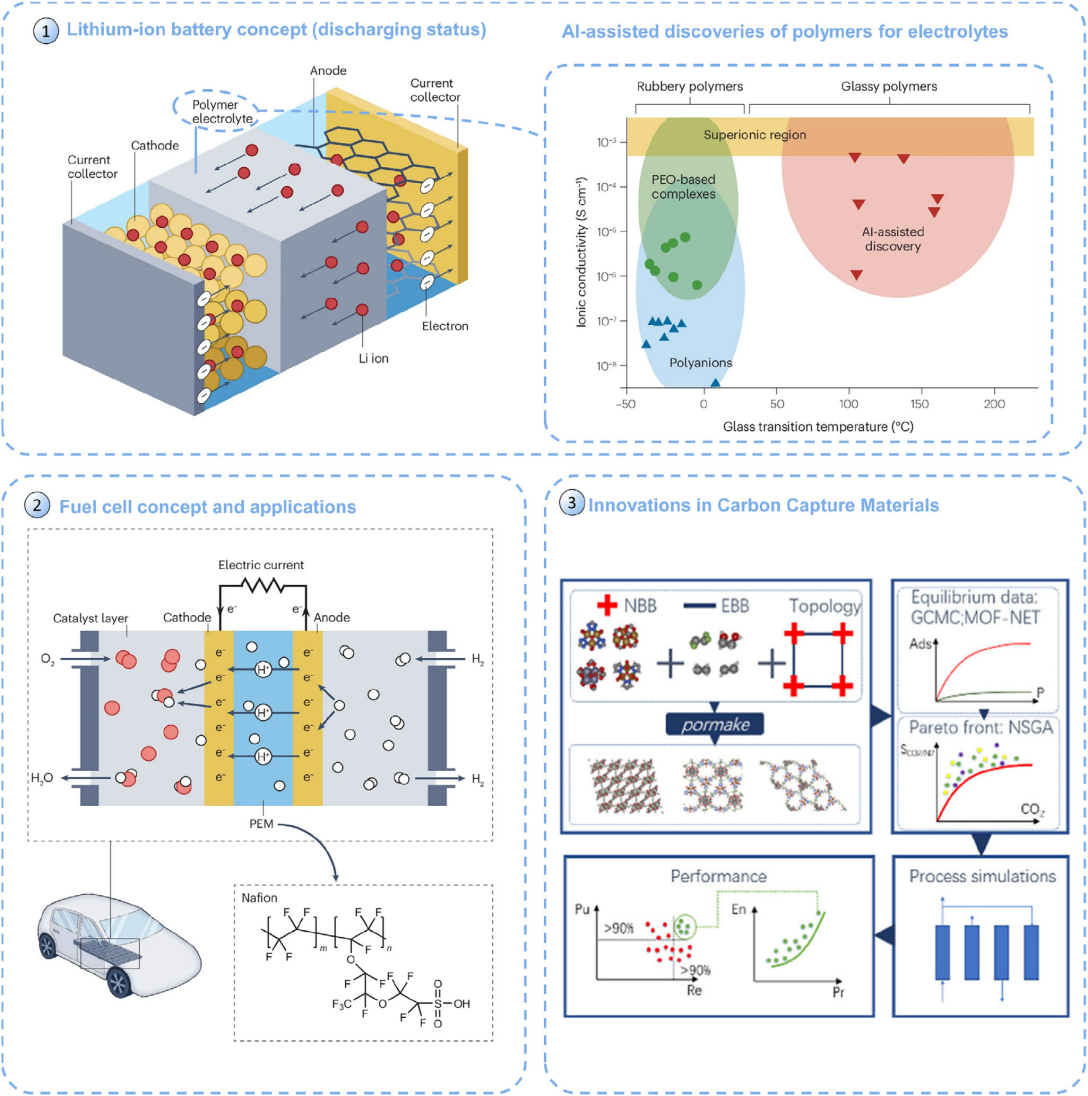
Overview of AI‐assisted materials design for energy and carbon capture. (1) Li‐ion battery components and an example of AI‐guided solid polymer electrolyte (SPE) discovery, illustrating ionic conductivity versus glass‐transition temperature. (2) A schematic illustration of a fuel cell, which often uses Nafion for a proton exchange membrane (PEM) and ionomers. Reprinted with permission from ref. [247], Nature Reviews Materials. (3) Workflow of metal–organic frameworks (MOFs) optimized for postcombustion carbon capture. Adapted from ref. [248], CC BY 4.0.

A Li‐ion battery cell (Figure 15, panel 1) consists of an anode and a cathode that reversibly (de)intercalate Li ions, an electrolyte enabling ionic transport, and a separator that prevents electrical shorting while allowing ion flow. Wang's thesis [249] curated datasets for doped spinel, doped NCM layered cathodes, and carbon‐coated doped olivine systems, and evaluated machine‐learning regression models for predicting discharge performance; tree‐based ensemble models (e.g., gradient boosting) achieved strong predictive accuracy on these tasks [249]. From a sustainability perspective, end‐of‐life recycling is largely process‐determined: route comparisons highlight trade‐offs among pyrometallurgical, hydrometallurgical, and direct cathode recycling [250], recovered cathode‐derived phases can be upcycled into value‐added catalysts [251], and feasible processes have been reported for recovering valuable metals from spent NCM cathodes [252]. Together, these studies motivate integrating ML‐guided performance screening with recycling‐aware constraints and process considerations, rather than treating “recyclability” as a standalone intrinsic materials claim.

Beyond batteries, AI is increasingly used to design catalysts for low‐carbon chemical energy conversion and to mitigate degradation mechanisms such as coking. Sun et al. [253] combined ML‐predicted C–H dissociation barriers with experimental validation to identify high‐performing SAA methane‐cracking catalysts (e.g., Ir/Ni and Re/Ni), and demonstrated that mechanical vibration/ball milling can mitigate coking to extend catalyst lifetime [253].

In fuel‐cell design, ML has been increasingly applied to tasks ranging from device‐level modeling [254, 255] to polymer discovery [256]. As illustrated in Figure 15 (panel 2), Tran et al. [256] proposed an informatics‐based screening strategy that defines target criteria for PEMs and ionomers, trains ML models to predict key properties, and screens large polymer spaces to identify promising candidates for downstream validation as potential Nafion alternatives [256].

**Adjacent Directions: Self‐Powered Sensing and Low‐Energy Wearable Interfaces**. Beyond storage and conversion, sustainability can also be improved at the *use phase* by self‐powered sensing and low‐power interfaces that reduce battery dependence and maintenance burdens. Representative examples include triboelectric nanogenerator (TENG) platforms for wearable respiratory monitoring and ML‐assisted TENG sensing for intelligent sports, as well as flexible electrochromic devices enabling low‐energy wearable visual feedback [257, 258, 259, 260]; complementary circular‐economy and closed‐loop recycling studies highlight end‐of‐life considerations for flexible electronics and e‐textiles [261, 262].

### 6.2 Multi‐Modal AI Driven Innovations in Carbon Capture Materials

In addressing the growing issue of carbon emissions, alongside afforestation and natural purification, carbon capture technology for $CO_{2}$ absorption has emerged as an effective solution. However, the cost and scale‐up of carbon‐capture systems and sorbent materials remain major barriers to wide deployment.As promising adsorbents for post‐combustion ${\mathrm{CO}}_{2}$ capture, metal–organic frameworks (MOFs) (Figure 15, panel 3) require improved regenerability (cyclic stability), robustness to realistic flue‐gas conditions (e.g., humidity), and process‐relevant performance.Growing materials databases and ML models enable accelerated screening and design of MOFs, and can be coupled with process‐level evaluation (e.g., ${\mathrm{CO}}_{2}$ purity and recovery in PSA/VSA cycles) to identify practically promising candidates.

Research by Andrzej Gładysiak's team [263] investigates MOFs' environmental adaptability. Using Al‐TCPB as the base MOF, they compared its performance with functional materials modified by hydroxyl or amino groups in carbon capture.All three materials showed strong cyclic stability under humid breakthrough and regeneration tests, with the hydroxyl‐functionalized variant Al‐TCPB(OH) outperforming the amino‐functionalized and pristine analogues under the reported conditions.Their DFT simulations provides mechanistic insights into adsorption in humid streams, which can inform future data‐driven screening and model development. Overall, the study combines macroscopic breakthrough cycling with microscopic characterization and DFT to build a multi‐modal evidence chain for regenerable ${\mathrm{CO}}_{2}$ capture materials. From a generative‐design perspective, Park et al. [264] developed an AI framework to construct MOF candidates with high ${\mathrm{CO}}_{2}$ uptake, followed by screening for synthesizability and structural stability.They proposed a diffusion‐model‐based generative framework to design MOF linkers and assemble candidate MOFs, followed by stability screening (MD) and adsorption evaluation using graph neural networks and GCMC simulations. The workflow identified a small set of AI‐generated MOFs with high ${\mathrm{CO}}_{2}$ uptake under the evaluated conditions, while MD‐based tests indicated limited lattice‐parameter changes, supporting both strong adsorption performance and structural robustness. In addition to screening, Deng and Sarkisov [248] proposed a multi‐scale workflow that assembles hypothetical MOFs from building blocks and topologies (e.g., using the PORMAKE constructor), applies ML‐guided evolutionary/multi‐objective optimization to prioritize candidates with favorable ${\mathrm{CO}}_{2}$/$N_{2}$ separation characteristics, and evaluates shortlisted materials via process‐level simulations of a modified Skarstrom cycle to assess practical separation performance.

Carbon capture materials mitigate emissions by separating ${\mathrm{CO}}_{2}$ from gas streams; the captured ${\mathrm{CO}}_{2}$ can then be stored in geological formations or utilized downstream (e.g., chemical production or mineralization), depending on the CCUS pathway. Once regenerable and durable carbon capture materials gain wider application, they could advance environmental protection and carbon‐related synthetic industries.

## 7 Data Integration, Challenges, and Future Perspectives

**FIGURE 16:**
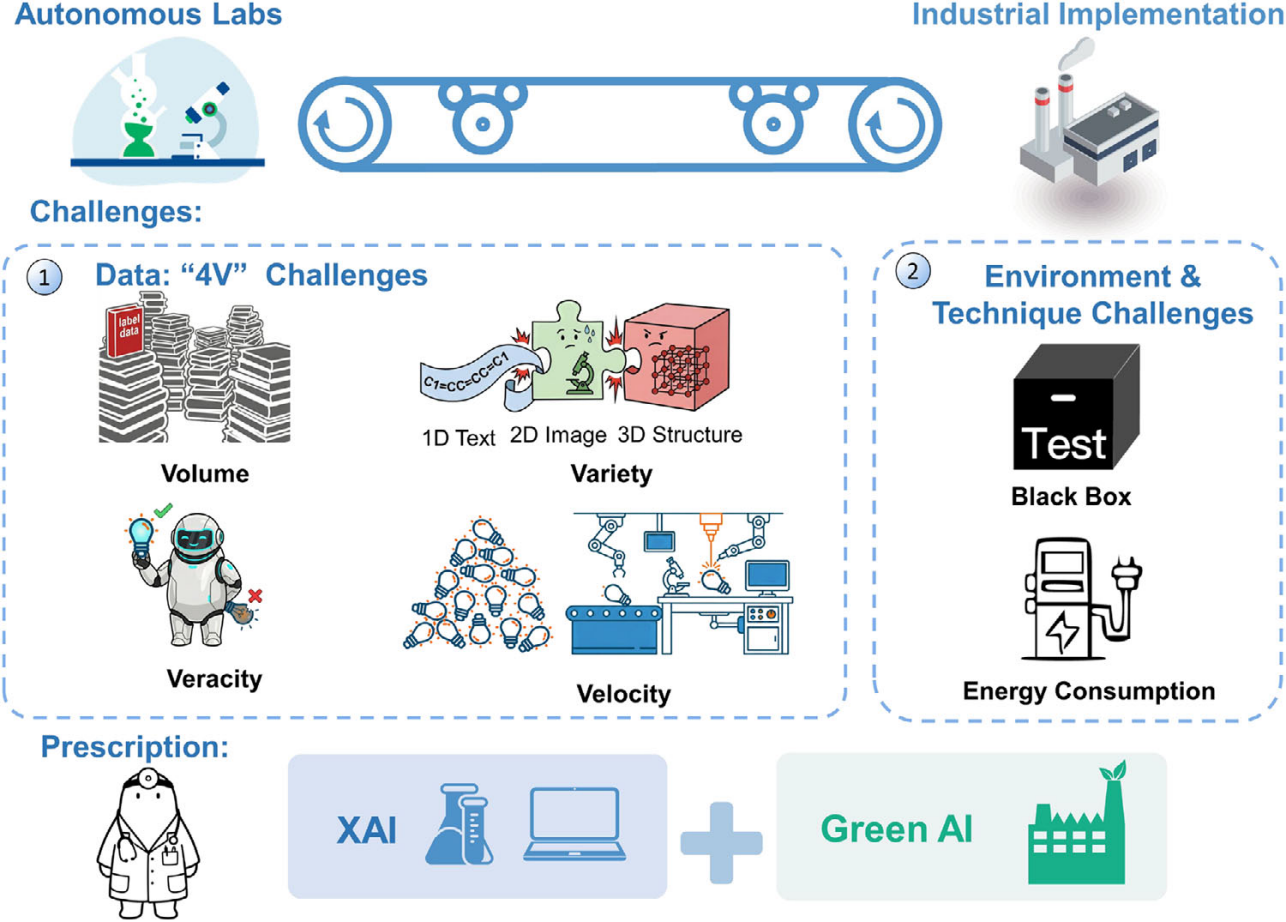
Data Integration, Challenges, and Future Perspectives. The framework illustrates the transition from **Autonomous Labs** to **Industrial Implementation**. (1) The Data Landscape is characterized by the “4Vs,” where aligning heterogeneous multi‐modal data (Variety) is foundational. (2) Challenges and Enablers: To address the opacity of “Black Box” models and high energy consumption, Explainable AI (XAI) and Green AI are deployed as critical mechanisms. These paradigms facilitate Sim‐to‐Real Validation, bridging the gap between theoretical prediction and scalable manufacturing.

To fully unleash the immense potential of multi‐modal AI in sustainable materials design, we must confront and systematically address a series of profound challenges [1]. These challenges are not only technical but also extend to data infrastructure, scientific methodology, and even ethical and environmental dimensions. This chapter will delve into these critical issues and look ahead to future trends, emphasizing eXplainable AI (XAI) and Green AI as the two cornerstones for the sustainable development of this field. XAI aims to transform “black‐box” predictors into genuine tools for scientific discovery, thereby addressing the sustainability of scientific knowledge itself [265]. Green AI, by focusing on the energy efficiency of the computational processes, ensures the environmental and economic sustainability of the field [266]. As illustrated in Figure 16, they form the indispensable components of a responsible and mature AI for Materials paradigm [1, 267]. For reference, we summarize commonly used materials benchmarks and their standard evaluation metrics in the Appendix (Table A2; metric glossary in Table A3), together with selected sustainability/workflow‐level evaluation protocols (Table A4).

### 7.1 The Data Landscape: Challenges in Multi‐Modal Materials Data Collection

Materials discovery data are inherently multi‐modal and multi‐scale, which presents the typical “4V” challenges of the big data era [268, 269, 270]:

- **Volume**: High‐throughput experiments and computations (such as combinatorial materials discovery) can generate massive amounts of raw data, the scale of which often exceeds the capacity for human analysis [271].
- **Velocity**: The advent of autonomous laboratories is causing an exponential increase in the speed of data generation, requiring data processing and decision‐making systems to have near‐real‐time response capabilities [237].
- **Variety**: Data sources and formats are extremely diverse, encompassing multiple modalities from unstructured text, 2D / 3D images, spectra, and crystal structures to numerical properties. This heterogeneity is a core challenge for multi‐modal fusion [47].
- **Veracity**: Data quality is inconsistent, with widespread issues of noise, missing values, inconsistent recording of experimental conditions, and conflicts between data from different sources, which places high demands on the robustness and reliability of models [270].

Currently, the materials discovery field severely lacks unified data standards, metadata specifications, and shared ontologies [14]. A vast amount of valuable data is “trapped” in the supplementary materials of papers, proprietary instrument file formats, or inconsistently formatted internal databases. This “data silo” phenomenon greatly hinders the aggregation, reuse, and cross‐validation of large‐scale data, making it extremely difficult to build general and powerful AI models [272]. Particularly for certain emerging material classes or specific properties, the scarcity of high‐quality, well‐annotated experimental data is a major bottleneck for training reliable AI models [46]. To address data scarcity, researchers are actively exploring data‐efficient learning methods, such as few‐shot learning, transfer learning, and self‐supervised learning, in the hope of training high‐performance models on limited data  [273].

### 7.2 Ethical, Environmental, and Technical Challenges

#### 7.2.1 Technical and Scientific Challenges: From Prediction to Understanding

Many advanced machine learning models (such as deep neural networks and ensemble tree models), while highly accurate in their predictions, have opaque internal decision‐making processes, resembling a “black box” [274]. This severely hinders scientists' trust and adoption of the models and, more critically, limits the potential to gain new scientific insights from them. eXplainable AI (XAI) technologies aim to open this “black box,” transforming the models' predictions into human‐understandable insights [275]. Post‐hoc explanation methods like SHAP (SHapley Additive exPlanations) [276] and LIME (Local Interpretable Model‐Agnostic Explanations) [277] can quantify the contribution of each input feature to a single prediction. The application of XAI is not just for building trust but for driving scientific discovery. For example, in predicting the Curie temperature of magnetic materials, SHAP analysis has enabled researchers to identify key physicochemical features, such as average magnetic moment and atomic number, that influence the prediction results, thereby linking the model's predictions with physical intuition and deepening the understanding of the material's intrinsic laws [278].

A reliable AI model must not only make accurate predictions but also “know when it is likely to be wrong.” Therefore, Uncertainty Quantification (UQ) is crucial [279]. Models need to be able to provide a confidence interval for their predictions, which is especially important in high‐stakes decisions (such as choosing expensive experimental protocols or deploying materials in safety‐critical applications). Furthermore, how to make models generalize to new material systems that are chemically very different from the training data (i.e., out‐of‐distribution prediction or extrapolation) remains a major unresolved challenge [280]. This is key to achieving true “discovery” rather than just “optimization.”

#### 7.2.2 OOD Generalization and Verification‐Centric Discovery

A central obstacle to discovery (rather than interpolation‐style optimization) is robust decision making under distribution shift: many sustainability‐relevant targets lie at the boundary of existing datasets, where compositions, defects, processing routes, recycled‐feedstock variability, or microstructures are poorly represented [133]. In such regimes, models may produce plausible‐looking but physically invalid or practically infeasible suggestions unless uncertainty, constraints, and verification are explicitly enforced [281, 282].

We therefore emphasize a *propose–verify* paradigm for sustainability‐oriented materials design. In this view, generative models and LLM‐based planners serve primarily as proposal generators; reliability is achieved by coupling proposals with calibrated uncertainty estimation, executable physical or chemical constraints (for example, charge neutrality, stoichiometry, and symmetry constraints), and tool‐mediated verification using high‐fidelity simulation and/or targeted experiments, ideally in closed‐loop settings [2, 111, 130]. Agentic workflows operationalize this paradigm by enabling iterative refinement with tool use and self‐correction, for example by repeatedly planning, retrieving evidence or running simulations, checking consistency, and revising decisions, which is difficult to achieve with a single closed forward pass [97, 167, 283].

Emerging directions that aim to learn more general internal representations, including predictive‐coding or world‐model style objectives (for example, JEPA‐like ideas) and neurosymbolic integration, may further improve robustness and counterfactual reasoning, but remain nascent in materials and are most compelling when combined with constraints and verification [135].

Finally, even as models scale, OOD failure modes (e.g., surfaces, defects, and rare events) remain a practical concern; reliable sustainability conclusions still require uncertainty‐aware validation and provenance‐aware knowledge integration, for example via retrieval‐augmented approaches when appropriate [100, 133]. Recent work has reported empirical neural scaling behavior in materials and chemical models, where average predictive loss improves approximately as a power law with increased data or model capacity (e.g., in large‐scale crystal discovery and in large chemical or atomistic models) [1, 29, 284]. Such trends suggest that continued scaling can improve mean accuracy and broaden applicability, but they do not eliminate distribution‐shift risks: OOD regimes (e.g., surfaces, defects, and activated rare events) can still exhibit qualitatively wrong physics even when in‐distribution metrics improve. We therefore refrain from over‐claiming “emergent” reliability for sustainability conclusions; instead, we advocate scaling together with uncertainty diagnostics, constraint enforcement, and targeted high‐fidelity verification in a propose‐verify loop.

#### 7.2.3 Ethical and Environmental Considerations: Responsible AI Innovation

The performance of AI models is highly dependent on their training data [46]. If historical research data is itself biased (for example, overly focused on certain “star” elements, common crystal structures, or experimental data from specific geographic regions), then the trained AI model is likely to learn and amplify these biases [285]. This could lead the model to systematically overlook regions of the chemical space that, while having great potential, have been historically under‐researched, thereby stifling innovation and potentially perpetuating a reliance on certain critical or unsustainable materials [47]. Therefore, developing techniques to detect, quantify, and mitigate data and model bias is crucial.

Meanwhile, training large‐scale AI models, especially large language models and generative models, requires enormous computational resources, and their energy consumption and carbon footprint cannot be ignored [286]. This presents a potential paradox: the tools we use to seek sustainable solutions may themselves be unsustainable. To address this challenge, the emerging field of “Green AI” has been established [266]. It is dedicated to developing more energy‐efficient and effective AI technologies at multiple levels, from algorithms (such as designing more lightweight model architectures) and hardware (such as developing more energy‐efficient computing chips) to data center operations (such as using renewable energy for model training) [287].

### 7.3 The Path to Industrial Implementation

While current materials informatics heavily relies on lab‐scale data—such as intrinsic properties, microstructures, and basic sustainability indicators [24, 47]—true materials sustainability necessitates a holistic life‐cycle perspective. To properly evaluate and design sustainable materials, data from diverse sources spanning industrial manufacturing and processing, end‐of‐life scenarios (e.g., recyclability, degradability), and health and environmental impacts (e.g., toxicity, emissions) are indispensable [288, 289]. However, acquiring this comprehensive life‐cycle data presents a formidable challenge. Unlike lab‐based properties that can be generated en masse via high‐throughput computations (e.g., density functional theory) or standardized experiments, life‐cycle data is notoriously scarce [290, 291, 292]. Much of the manufacturing and processing data remains proprietary to industries, creating a barrier to open research [293]. Furthermore, there is a significant “scale mismatch” between gram‐scale lab synthesis and ton‐scale industrial production, making the direct extrapolation of energy consumption and carbon footprints highly unreliable [294, 295]. Additionally, standardization remains a major challenge in life‐cycle‐based sustainability assessment, with substantial variation in system boundaries, inventory data, and methodological choices across studies [292, 296].

Even when such data becomes available, integrating it into existing AI / ML pipelines introduces profound algorithmic challenges [24, 47]. First, life‐cycle and environmental data are highly heterogeneous. An ML model must simultaneously process continuous numerical data (e.g., yield strength), high‐dimensional data (e.g., microscopy images), and categorical or text‐based environmental reports (e.g., Life Cycle Assessment documents) [109, 297]. Fusing these multi‐modal inputs requires novel neural network architectures. Second, there is a severe data imbalance; while thousands of data points might exist for a material's physical properties, only a handful might exist for its recycling cost or long‐term toxicity. This sparsity demands advanced techniques like multi‐fidelity modeling, transfer learning, or physics‐informed neural networks to prevent overfitting [298, 299]. Finally, incorporating sustainability transforms the AI task into a highly complex multi‐objective optimization problem. The AI must navigate vast chemical spaces to find Pareto‐optimal solutions that balance cutting‐edge functional performance with minimal carbon footprint, low toxicity, and economic viability [51, 55].

Beyond data and algorithmic complexities, translating these computationally optimized designs into physical reality poses another major hurdle. Whether a computationally designed material can be successfully synthesized in the real world and whether its performance matches predictions is a critical step for AI‐driven materials discovery to move toward application [287]. Autonomous laboratories, by rapidly iterating experiments to validate AI predictions, play an indispensable role in accelerating the “sim‐to‐real” cycle and bridging this gap [69].

Ultimately, the true integration of these AI technologies and autonomous platforms into industrial R&D processes still faces many practical obstacles. These include the urgent demand for talent with interdisciplinary knowledge (materials science + data science) [24], the high upfront investment in computational infrastructure and automation equipment [70], and the organizational resistance to changing deeply entrenched traditional R&D cultures and workflows [148]. In addition, data privacy and intellectual property issues also need to be properly addressed in a collaborative innovation ecosystem [68].

## 8 Conclusion

### 8.1 Summary of Key Insights: Enabling Sustainable Alloys through Multi‐Modal AI

This review has systematically elucidated how multi‐modal AI is invigorating sustainable materials design and catalyzing a new, efficient, and integrated R&D paradigm. The core progress can be summarized as follows: AI is reshaping the entire lifecycle of sustainable materials. At the design stage, inverse design informed by environmental proxies and selected life‐cycle indicators is becoming feasible, allowing environmental impact to be considered earlier in material creation.At the synthesis stage, autonomous laboratories can reduce trial‐and‐error and improve resource efficiency through closed‐loop optimization. During the application stage, AI can predict the service life and durability of materials. At the end‐of‐life stage, intelligent sorting technologies provide efficient solutions for material recycling. Taken together, these advances point toward a more closed‐loop and sustainability‐aware materials innovation ecosystem, marking a shift in materials science from traditional, linear exploration focused on a single performance goal to a multi‐objective, full‐lifecycle optimization centered on sustainability.

### 8.2 Future Directions: Towards Widespread Adoption and Impact

Looking ahead, a “Materials‐on‐Demand” ecosystem is emerging. In this ecosystem, a researcher or engineer could issue a complex, multi‐constraint material request to a multi‐agent AI system in natural language. Upon receiving the instruction, this AI system would autonomously and collaboratively perform the following tasks:

**Task Decomposition and Knowledge Retrieval**: A “planner” agent would break down the complex request into a series of executable sub‐tasks. Simultaneously, multiple “knowledge retrieval” agents would leverage large language models to mine global scientific literature, patent databases, and material property databases to synthesize existing knowledge, forming an initial design space and set of constraints.

**Candidate Material Generation and Virtual Screening**: Using multi‐modal generative models (such as diffusion models or GANs), a “creator” agent would design thousands of novel, high‐potential candidate material structures. Subsequently, a “screener” agent would use high‐throughput virtual screening with rapid surrogate models to predict the comprehensive performance of these candidates (including target properties, cost, LCA metrics, and synthetic feasibility), selecting the few most optimal candidates.

**Autonomous Experimental Planning and Execution**: An “experiment planner” agent would design detailed synthesis and characterization protocols for the optimal candidates. This plan would then be sent to a globally networked “Cloud Lab,” where robotic systems would automatically complete the material synthesis, characterization, and performance validation.

**Multi‐modal Data Analysis and Iteration**: An “analyst” agent would perform real‐time analysis of the multi‐modal experimental data transmitted back from the cloud lab (such as microscopic images, spectra, performance curves, etc.) and feed the results back to the “planner” agent. The system would automatically update its internal models based on the deviation between experimental results and initial predictions, and could initiate a new design‐build‐test‐learn cycle until the target is met.

The realization of this vision will compress the new material discovery cycle from decades to months or even weeks, thereby revolutionizing technological innovation to address pressing global challenges such as climate change, energy crises, and public health.

### 8.3 A Call for Interdisciplinary Collaboration and Final Outlook

Achieving the grand vision described above is far beyond the capability of any single discipline. It urgently requires breaking down the traditional barriers between materials science, chemistry, computer science, robotics, physics, environmental science, and public policy. The complex challenges inherent in the grand theme of sustainability are, by their nature, interdisciplinary. multi‐modal AI, with its ability to fuse heterogeneous information and simulate complex decision‐making processes, is becoming the common language and core platform for promoting such interdisciplinary collaboration. The materials scientist of the future will need not only deep domain knowledge but also mastery of data science and AI tools, becoming a “centaur”‐like researcher capable of efficient collaboration with intelligent systems.

Ultimately, our goal is to build an open, collaborative, and cross‐disciplinary global research community. By sharing data, models, and automated experimental capabilities, we can fully unlock the potential of AI to jointly create a better future driven by sustainable new materials. This is not just a technological revolution but a profound transformation of scientific culture, one that will lead us into a new era where we can create material solutions rapidly, precisely, and responsibly, according to the needs of society.

## Author Contributions

Tianyi Xu, Tianshuo Wei and Yan Ge contributed equally to this work. Tianyi Xu, Tianshuo Wei and Yan Ge developed the detailed content of the review and drafted and revised the manuscript. Bo Peng, Maolin Wang and Yue Li contributed to manuscript drafting and revision, and provided guidance on the literature survey and analysis. Peng Wen, Chao Yang and Ye Wei conceived and supervised the overall project, provided strategic guidance throughout the review and contributed substantially to manuscript writing and critical revision.

## Conflicts of Interest

The authors declare no conflicts of interest.

## Appendix A

**TABLE A1:** LLM‐centric paradigms that can support sustainable materials design.

Method family	Modalities	Sustainability role / targets	What it enables	Limitations	Refs.
Literature and figure mining for synthesis / processing data	T, I, P, G	Builds structured evidence for footprint, yield, and recyclability constraints	Turns papers (text+figures) into databases of recipes / conditions / properties for downstream screening	Parsing ambiguity; reporting inconsistency; bias; needs validation	[150, 300, 301, 302]
Domain‐adapted LLMs and efficient adapters	T, P	Better materials / process reasoning for design and decision support	Improves domain language and task performance; supports process‐aware queries	Data quality / licensing; overfitting risk; still needs grounding / verification	[150, 157, 158, 159]
Alloy LMs for prediction and design under constraints	T, S, P, Sim	Faster candidate proposal with property + phase / feasibility constraints	Predicts properties, proposes candidates, answers phase‐diagram questions; supports constrained screening	Coverage / extrapolation; needs CALPHAD / DFT / experiments	[208, 209, 210, 303, 304]
Crystal structure generation + synthesis planning with LMs	S, T, Sim	Adds synthesis feasibility and route / precursor constraints	Generates structures; predicts synthesizability, routes, and precursors from structure strings	Representation sensitivity; dataset bias; lab validation required	[99, 305]
Retrieval‐grounded + tool‐using / multi‐agent workflows	T, S, P, Sim, G	Evidence‐grounded iterative discovery; can call sustainability databases and simulators	Plans, retrieves, calls tools/simulations, checks, and revises in loops	Failures cascade; compute cost; governance / safety; robustness hard to guarantee	[97, 166, 211, 306, 307]
Evaluation and benchmarks (knowledge, tools, images)	T, I, S, Sim	Reliability testing before deployment	Benchmarks domain knowledge, multi‐step reasoning, tool execution, and characterization images	Benchmark–practice gap; leakage; metric gaming; judge bias	[161, 308, 309]

**TABLE A2:** Representative benchmark tasks and standardized metrics used to evaluate AI approaches relevant to (multi‐modal) materials discovery.

Benchmark	Task	Typical methods	Metrics	Refs.
MatBench	Property prediction	RF / GNN / Transformer surrogates	Regression metrics: MAE / RMSE / MAPE; Classification metrics: ROC‐AUC / F1 / accuracy	[310]
Matbench Discovery	Crystal stability screening in HT discovery setting	Energy models; GNN / MLIP surrogates; ranking pipelines	Regression metrics: MAE / RMSE / $R^{2}$; Stability classification: F1 / TPR / TNR; Discovery under budget: DAF; precision/recall vs screening budget	[311]
Open Catalyst (OC20 / OC22)	Catalysis: structure → energy / forces; relaxation	Equivariant GNNs; MLIPs/uMLIPs	Energy error: Energy MAE; Force errors: Force MAE; Force Cosine; Threshold success: EFwT (Energy & Forces within Threshold)	[312]
CatBench	Adsorption‐energy prediction for catalysis workflows	Universal MLIPs / surrogates	Adsorption‐energy error: MAE (overall + normal subset); Reliability: normal rate; anomaly‐rate; Within‐threshold success: ADwT / AMDwT	[313]
Crystal generation (CDVAE)	Crystal generation + distribution matching	VAE / diffusion generators	Generation validity; Coverage metrics (COV‐R / COV‐P); Distribution distance (EMD on properties)	[44]
MatTools	LLM tool‐use for materials packages	LLMs (tool calling, code generation, agentic workflows)	Question answering: accuracy (MCQ); Executable tool‐use: FRR (runnable rate), TSR (task success rate)	[161]
MatCha	multi‐modal benchmark on characterization images	multi‐modal LLMs (VLMs)	Multiple‐choice accuracy; Stage‐wise accuracy (PC / MA / SA / PA)	[308]
FDM‐Bench	Additive manufacturing tasks	LLMs for diagnosis/troubleshooting; reasoning over G‐code + user queries	G‐code anomaly detection: accuracy; Confidence scoring (probability assigned to ground‐truth label); User queries: expert scores (1–5): accuracy/precision/relevance	[309]

**TABLE A3:** Metric for Table A2.

Metric	Explanation
MAE / RMSE	Regression errors measuring average absolute (MAE) or squared (RMSE) deviations between predictions and ground truth; lower is better.
ROC‐AUC / F1	Classification metrics; ROC‐AUC measures ranking quality across thresholds, and F1 balances precision and recall at a chosen threshold; higher is better.
TPR / TNR	True positive rate (recall) and true negative rate (specificity) in stability classification; higher is better.
Precision@k / Recall@k	Discovery under a screening budget: performance among the top‐k ranked candidates; higher is better.
DAF (Discovery Acceleration Factor)	Speed‐up of model‐guided screening over a baseline (typically random) for finding target materials under comparable budgets; larger indicates faster discovery.
Force Cosine	Directional similarity between predicted and reference force vectors in atomistic benchmarks; higher is better.
EFwT (Energy & Forces within Threshold)	Fraction of cases where both energy and force errors are below benchmark‐defined thresholds; higher is better.
ADwT / AMDwT (CatBench)	Within‐threshold success rates for adsorption‐energy prediction, summarizing the fraction of cases meeting benchmark‐defined error thresholds (including anomaly‐aware variants); higher is better.
Validity (generation)	Fraction of generated structures satisfying basic physical/structural constraints; higher is better.
COV‐R / COV‐P (coverage)	Coverage of reference by generated samples (and vice versa), capturing diversity and distributional overlap; higher is better.
EMD (Earth Mover's Distance)	Distance between generated and reference property distributions; lower is better.
FRR / TSR (tool‐use)	Runnable rate of tool / code outputs (FRR) and end‐to‐end task success rate (TSR) for tool‐using LLM workflows; higher is better.
Stage‐wise accuracy (MatCha)	Multiple‐choice accuracy reported separately for different research stages (e.g., comprehension / measurement / scientific analysis / planning) to localize failure modes.
Expert scores (FDM‐Bench)	Human ratings (typically 1–5) for open‐ended manufacturing queries (e.g., accuracy/precision/relevance), complementing objective anomaly‐detection accuracy.

**TABLE A4:** Sustainability‐ and workflow‐level evaluation protocols for comparing sustainable materials discovery pipelines beyond model‐level predictive accuracy.

Protocol / framework	Scope	What it measures	Outputs / metrics	Refs.
SIMS‐$D^{3}$	Sustainability‐informed materials selection, design, discovery, and development	Lifecycle‐ and hazard‐aware decision making under constraints (multi‐criteria sustainability assessment)	Multi‐indicator sustainability profiles (e.g., footprint‐style indicator sets); trade‐off analysis / Pareto‐style comparisons	[314]
XPIs	Evaluation of accelerated discovery workflows (platform‐level comparison)	System‐level gains from AI‐enabled discovery platforms (time, quality, resources, carbon, labor)	XPI‐1–XPI‐5 (component indices) + overall acceleration score	[315]
